# Anion-Exchange Strategy for Ru/RuO_2_-Embedded N/S-*Co*-Doped Porous Carbon Composites for Electrochemical Nitrogen Fixation

**DOI:** 10.3390/polym17040543

**Published:** 2025-02-19

**Authors:** Shahzeb Ali Samad, Xuanzi Ye, Zhiya Han, Senhe Huang, Chenbao Lu, Junbo Hou, Min Yang, Zhenyu Zhang, Feng Qiu, Xiaodong Zhuang

**Affiliations:** 1 The Soft2D Lab, State Key Laboratory of Metal Matrix Composites, Shanghai Key Laboratory of Electrical Insulation and Thermal Ageing, School of Chemistry and Chemical Engineering, Shanghai Jiao Tong University, 800 Dongchuan Road, Shanghai 200240, China; shahzeb@sjtu.edu.cn (S.A.S.); yxz134340@sjtu.edu.cn (X.Y.); sh.huang@sjtu.edu.cn (S.H.); castle@sjtu.edu.cn (C.L.); 2 School of Materials, Shanghai Dianji University, 300 Shuihua Road, Pudong New Area District, Shanghai 201306, China; zhiyahan@sdju.edu.cn (Z.H.); 32133@sdju.edu.cn (M.Y.); 3 Power System Resources Environmental Technology Co., Ltd., 585 Changan North Road, Jiaxing 314399, China; junbo.hou@yahoo.com; 4 Shanghai Nuclear Engineering Research and Design Institute Co., Ltd., 169 Tianlin Road, Xuhui District, Shanghai 200030, China; 5 School of Chemical and Environmental Engineering, Shanghai Institute of Technology, 100 Haiquan Road, Shanghai 201418, China; 6 Frontiers Science Center for Transformative Molecules, Zhang Jiang Institute for Advanced Study, Shanghai Jiao Tong University, 429 Zhangheng Road, Shanghai 201203, China

**Keywords:** anion-exchange method, ionic covalent organic framework, porous carbon, ruthenium nanoparticle, nitrogen reduction reaction

## Abstract

Ionic porous polymers have been widely utilized efficiently to anchor various metal atoms for the preparation of metal-embedded heteroatom-doped porous carbon composites as the active materials for electrocatalytic applications. However, the rational design of the heteroatom and metal elements in HPC-based composites remains a significant challenge, due to the tendency of the aggregation of metal nanoparticles during pyrolysis. In this study, a nitrogen (N)- and sulfur (S)-enriched ionic covalent organic framework (*i*COF) incorporating viologen and thieno[3,4-b] thiophene (TbT) was constructed via Zincke-type polycondensation. The synthesized *i*COF possesses a crystalline porous structure with a pore size of 3.05 nm, a low optical band gap of 1.88 eV, and superior ionic conductivity of 10^−2.672^ S cm^−1^ at 333 K, confirming the ionic and conjugated nature of our novel iCOF. By applying the iCOF as the precursor, a ruthenium and ruthenium(IV) oxide (Ru/RuO_2_) nanoparticle-embedded N/S-co-doped porous carbon composite (NSPC-Ru) was prepared by using a two-step sequence of anion-exchange and pyrolysis processes. In the electrochemical nitrogen reduction reaction (eNRR) application, the NSPC-Ru achieves an impressive NH_3_ yield rate of 32.0 μg h^−1^ mg^−1^ and a Faradaic efficiency of 13.2% at −0.34 V vs. RHE. Thus, this innovative approach proposes a new route for the design of *i*COF-derived metal-embedded porous carbon composites for enhanced NRR performance.

## 1. Introduction

Ammonia is one of the most essential chemical products and has been widely used in the fields of agricultural fertilizers, industrial chemicals, and energy carriers globally. Owing to the chemical inertness of nitrogen molecules, the traditional industrial technology for producing ammonia is the Haber–Bosch process, which combines nitrogen (N_2_) and hydrogen (H_2_) to form ammonia (NH_3_); however, it suffers from a low conversion efficiency of ~10–15%, harsh reaction conditions with high temperature and pressure, and serious pollution [1,2]. The electrocatalytic nitrogen reduction reaction (eNRR) has attracted more attention in recent years due to its mild operation at ambient temperature and pressure without emitting carbon dioxide, offering a renewable and eco-friendly alternative for ammonia production when it combines green electronic sources (solar, wind, and tidal energy) for power [3,4]. The electrocatalyst is the key component in the eNRR system, which is utilized for the construction of ammonia-producing electrochemical cells with high efficiency and stability. Many metals have been applied for the eNRR with good NH_3_ yield. The modification of metals can improve NRR performance via defect engineering, heteroatom doping, and the optimization of the electronic structure of the catalysts [5,6,7]. Currently, various carbon materials, like graphene, carbon nanotube, and porous carbon, have been used as the conductive supports to prepare carbon composites, confirming a promising approach to the design of electrocatalysts. Furthermore, the intrinsic electronic properties of carbon materials could be effectively adjusted by the incorporation of different heteroatoms, including N, S, boron (B), and phosphorus (P), into the skeleton of carbons, resulting in their enhanced electrocatalytic activity in the NRR [8,9,10,11]. Unfortunately, the tendency of the aggregation of metal nanoparticles would severely reduce the exposure of the active site of electrocatalysts, resulting in their limited electrocatalytic application. Thus, the rational design of metal/porous carbon composites with a uniform distribution of metal atoms and nanoparticles would be highly desirable for NRR applications.

Porous polymer-derived carbons are highly structured with large surface areas, making them ideal supports for electrocatalysts [12,13,14,15]. The mesoporous structure of porous polymer allows porous polymer-derived carbons to effectively host active nanoparticles. Moreover, heteroatoms can be incorporated into the framework of porous carbons by using various heteroatom-containing molecules as the building blocks. Covalent organic frameworks (COFs), introduced by Yaghi et al. in 2005, are a kind of crystalline porous polymers linked by reversible covalent bonds [16]. COFs possessing defined porous structures and high specific surface areas have been applied as precursors for the construction of different porous carbon materials. However, most COFs have been constructed from neutral building blocks, limiting their interactions with metal ions; therefore, nanosized metal nanoparticles are formed to aggregate easily during high-temperature pyrolysis [17,18,19]. Recently, ionic COFs (*i*COFs) are the ideal precursors for the construction of metal/porous carbon composites. *i*COFs can be tailored as either anionic or cationic by incorporating ionic monomers or modifying neutral COFs post-synthesis. Their well-organized porous channels facilitate efficient ion transport, enabling counter-charged ions to move selectively and rapidly. This controlled ion conduction is crucial to energy-related applications. In particular, cation–π interactions, where cations interact with π-electron-rich systems like graphene, play a significant role in enhancing charge transport and stability, making them highly relevant to electrochemical processes [20,21]. Conventional approaches to synthesizing *i*COFs typically involve either preparing ionic building blocks or post-functionalizing COFs with ionic groups. These methods often demand complex organic reactions and exhibit low reaction conversion efficiency [22,23,24,25]. To date, the direct synthesis of *i*COFs from ionic linkages remains a significant challenge. More importantly, the exploitation of these *i*COFs for the preparation of metal/porous carbon composites and their electrocatalytic applications have still rarely been addressed.

Viologens, 4,4′-bipyridinium complexes, are a series of ionic conjugated molecules with rich redox chemistry. Many viologen-based *i*COFs have been effectively created via one-step Zincke reaction synthesis [26,27,28]. In this work, we present a novel viologen-based *i*COF-Cl containing TbT as the building block, in which viologen and TbT were used as the N and S sources. The as-prepared *i*COF-Cl shows a crystalline structure with a pore size of 3.05 nm, a low band gap of 1.88 eV, and ionic conductivity of 10^−2.672^ S cm^−1^ at 333 K. After anchoring [Ru(CN)_6_]^4−^ complexes onto the *i*COF-Cl by the ion-exchange method, the *i*COF-Ru could be turned into an NSPC-Ru with a uniform distribution of Ru/RuO_2_ nanoparticles via a pyrolysis treatment. As the electrocatalyst, the NSPC-Ru exhibited excellent electrochemical NRR performance, including an NH_3_ yield rate of 32.0 μg h^−1^ mg^−1^ and a Faradaic efficiency of 13.2% at −0.34 V vs. RHE. This proof-of-concept study provides a solid foundation to develop metal-embedded heteroatom-doped porous carbon composites for electrochemical NRR applications.

## 2. Materials and Methods

### 2.1. Materials

2,4-Dinitrochloro benzene, thieno[3,2-b]thiophene, N-bromosuccinimide (NBS), dimethyl formamide (DMF), palladium tetrakis(triphenyl)phosphine (9.2% (Pd) RG), ethanol (≥99.7% AR), tetrahydrofuran (99% RG), hexane (97% AR), acetone (99.5% HPLC), dichloromethane (≥99.5% AR), chloroform (≥99% AR), 4,4′-bipyridine (98% RG), 1-chloro-2,4-dinitrobenzene (98% RG), nitrogen (N_2_) (99.99% AR; Air Liquefied Group), argon (Ar), potassium sulphate (K_2_SO_4_), ammonium sulfate ((NH_4_)_2_SO_4_), salicylic acid, sodium citrate, sodium hypochlorite (NaClO), and sodium nitroprusside dihydrate (Na_2_[Fe(CN)_5_NO]·2H_2_O) were used. None of the above-mentioned reagents required post-treatment before use.

### 2.2. Instruments

Nuclear magnetic resonance (NMR) spectra in liquid phase were captured by using a Brucker AVANCE III HD (500 MHz) spectrometer, with tetramethylsilane serving as the internal reference. All chemical shifts are reported in ppm relative to the signals corresponding to the residual non-deuterated solvents (D_2_O, *δ* = 4.97 ppm; DMSO-*d*_6_, *δ* = 2.50 ppm; CDCl_3_, *δ* = 7.26 ppm). Coupling constant values (J) are given in hertz (Hz), and multiplicity is abbreviated in the following way: s (singlet), d (doublet), t (triplet), and splitting patterns that were not easily interpretable were labeled as multiplet (m). Mass spectrometry via MALDI-TOF was conducted by using an Autoflex Speed TOF/TOF Matrix-assisted laser desorption ionization time-of-flight mass spectrometer. Fourier transform infrared (FT-IR) spectroscopy was carried out by using a spectra 100 spectrometer from Perkin Elmer, Inc., Springfield, IL, USA. Measurements on AXIS Ultra DLD were conducted for XPS analysis. The thermal stability of all samples in a nitrogen atmosphere was investigated by performing TGA on a Discovery TGA550 thermogravimetric analyzer. A S 2150 Hitachi Corp (Japan) was used for SEM analysis. HRTEM images were obtained by using a FEI Sirion, 200. UV–vis measurements were taken at room temperature by using a Lamda-950. The UPS spectra were obtained by using an ESCALAB250Xi instrument (from Thermo Fisher Scientific, Waltham, MA, USA) with a monochromatic He light source (21.22 eV).

### 2.3. Preparation Procedure

Preparation of 2,5-dibromothieno[3,2-b] thiophene (Th-Br): The synthesis of Th-Br was carried out according to previously reported methods [29]. To a well-stirred solution of thieno[3,2-b] thiophene (TbT) (3.5 g, 25 mmol) in CHCl_3_ (75 mL), NBS (8.9 g, 50 mmol) dissolved in DMF (50 mL) was added dropwise at 0 °C in the absence of light. The mixture was then allowed to warm slowly to room temperature. After stirring for 12 h, the reaction was quenched with ice, and the aqueous phase was extracted three times with DCM. The combined organic layers were washed several times with brine and dried over MgSO_4_, and the solvent was removed under reduced pressure to give pale yellow flakes of Th-Br. Yield: 7.0 g (94%). The product should be stored at −20 °C in a refrigerator to prevent decomposition into a black solid; ^1^H NMR (500 MHz, DMSO-*d*_6_, *δ*): 7.60 (*s*, 2H); ^13^C NMR (101 MHz, DMSO-d_6_, *δ*): 138.6, 123.4, 113.5; MALDI-TOF *m*/*z*: [M + H]^+^ calcd. for C_6_H_2_Br_2_S_2_, 297.79; found, 297.48.

Preparation of 2,5-di(pyridin-4-yl) thieno[3,2-b] thiophene (ThBiPy): The synthesis of ThBiPy was carried out according to previously reported methods with some modifications [30]. A 250 mL oven-dried two-necked round-bottom flask was cooled in a nitrogen atmosphere and charged with Th-Br (2.0 g, 6.71 mmol), 4-pyridinylboronic acid (2.47 g, 20.13 mmol), Pd (PPh_3_)_4_ (0.38 g, 0.335 mmol), powdered NaOH (1.51 g, 26.84 mmol), and 100 mL of a toluene/EtOH/H_2_O mixture (3:2:1 *v*/*v*). The reaction mixture was heated to reflux in a nitrogen atmosphere for 2–3 days. The completion of the reaction was indicated by a color change from yellow to dark brown and confirmed by TLC analysis. The reaction mixture was then cooled to room temperature, and the solvents were removed under reduced pressure. The solid residue was extracted with CH_2_Cl_2_ and washed with a brine solution. The organic phase was dried over anhydrous Na_2_SO_4_ and concentrated in vacuo. The pure product was isolated by silica gel column chromatography by using a CH_2_Cl_2_/EA/1–5% MeOH mixture as the eluent, yielding ThBiPy as an orange solid. Yield: 1.53 g (77%); ^1^H NMR (500 MHz, DMSO-d_6_, δ): 7.71 (d, 4H), 8.28 (s, 2H), 8.63 (d, 4H); ^13^C NMR (500 MHz, DMSO-d_6_, δ): 151.0, 144.8, 131.8, 129.2, 121.6, 119.9; MALDI-TOF *m*/*z*: [M + H]^+^ calcd for, 294.39; found, 294.13.

Synthesis of 4,4′-(thieno[3,2-b] thiophene-2,5-diyl)) bis(1-(2,4-Dinitrophenyl)-4,4′-bipyridilium dichloride (Zincke-ThBiPy): The synthesis of Zincke-ThBiPy was carried out according to previously reported methods [31]. ThBiPy (40 mmol) and 2,4-dinitrochlorobenzene (100 mmol) were refluxed in ethanol (250 mL) for 16 h. After cooling, the precipitate was filtered and washed with acetone to obtain Zincke-ThBiPy. Yield: 90%. ^1^H NMR (400 MHz, D_2_O, δ): 8.86 (d, 2H), 8.47 (d, 2H), 8.19 (d, 2H), 8.94 (d, 4H), 9.31 (d, 4H), 8.50 (s, 2H). MALDI-TOF *m*/*z*: [M − Cl]^+^ calcd for C_28_H_16_N_6_O_2_S_22_, 628.59; found, 628.03.

Preparation of ionic two-dimensional covalent organic framework (iCOF-Cl): The ionic 2D-COF was prepared by using according to a method reported previously, but the reported method failed to achieve the 2D-COF, while we acquired the material with some modifications to the method [26]. An EtOH/water (5:5 *v*/*v*) mixture of 1,3,5-tris(4-aminophenyl) benzene (0.5 mmol, 0.175 g) and Zincke-ThBiPy was degassed by three freeze–pump–thaw cycles in a sealed tube (25 mL). The tube was kept in the oven for three days, with the temperature being maintained at 100 °C, and subsequently cooled to room temperature. The orange powder formed was washed with ethanol twenty times to purify the product and dried. The remaining solid product was then immersed in acetone overnight and then dried in oven at 60 °C to obtain the *i*COF-Cl.

Preparation of ionic two-dimensional COF covered with [Ru(CN)_6_]^4−^: In a standard anion-exchange process, 1.0 g of *i*COF-Cl was first dispersed in 10 mL of water and subjected to 30 min of ultrasonication to ensure even distribution. An aqueous solution of K_4_[Ru(CN)_6_] (10 mg in 5 mL of water) was then gradually added to the dispersion while stirring. This mixture was stirred at room temperature for 24 h. After this period, 5 mL of the dispersion was filtered, and the remaining powder was returned to the solution, with another portion of K_4_[Ru(CN)_6_]solution being added slowly. This cycle was repeated daily for seven days to achieve complete anion exchange. Following the final filtration, the product was washed three times with deionized water and freeze-dried to yield the [Ru(CN)_6_]^4−^-exchanged *i*COF-Cl, designated as *i*COF-Ru. This thorough process ensures effective anion exchange and results in a stable and functionalized material.

Preparation of Ru-RuO_2_-anchored NPSC-Ru by pyrolysis: The as-prepared *i*COF-Ru was subjected to high temperature at 900 °C for 2 h, with the temperature rising at a rate of 10 °C per minute, resulting in the final NPSC-Ru product.

## 3. Results

**Figure 1:**
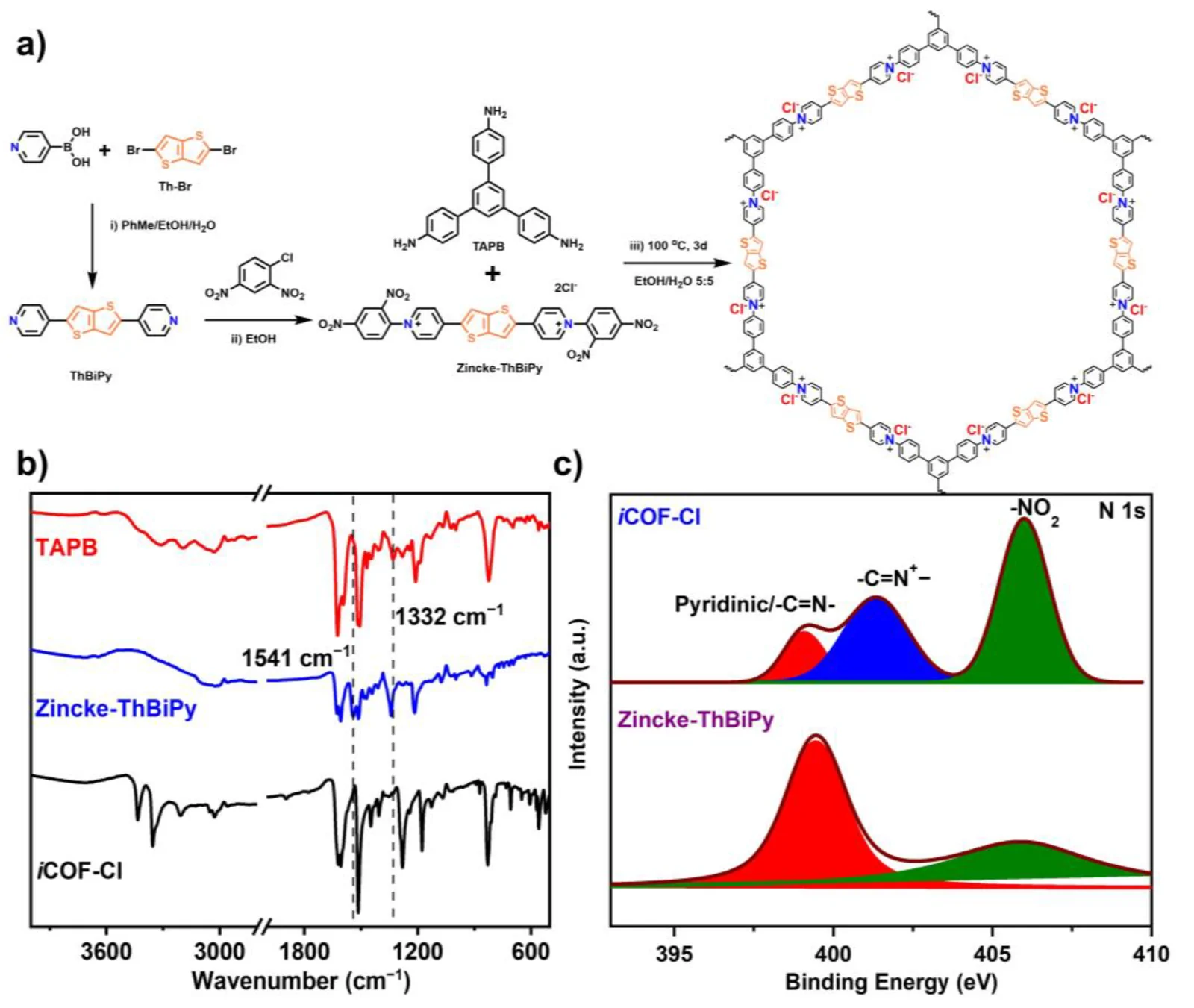
(**a**) Synthesis of *i*COF-Cl: (i) reflux, N_2_, 2 days; (ii) reflux, N_2_, 16 h; and (iii) 100 °C, N_2_, 3 days; thieno[3,2-b] thiophene unit, pyridinium and counter anion present orange, blue and red color, respectively. (**b**) FT-IR spectra of TAPB, Zincke-ThBiPy, and *i*COF-Cl. (**c**) N 1s spectra of *i*COF-Cl and Zincke-ThBiPy.

The synthesis route to the *i*COF-Cl is given in Figure 1a. The key intermediate of 2,5-di(pyridin-4-yl) thieno[3,2-b] thiophene (ThBiPy) was synthesized through a two-step procedure. First, a Suzuki cross-coupling reaction between pyridin-4-ylboronic acid and 2,5-dibromothieno[3,2-b] thiophene yielded the π-conjugated precursor. Subsequently, the resulting compound underwent a Zincke reaction with 1-chloro-2,4-dinitrobenzene to produce Zincke-ThBiPy in the yield of 90%. Then, Zincke-ThBiPy was reacted with tris(4-aminophenyl) benzene (TAPB) to form the ionic covalent organic framework *i*COF-Cl in the mixture of ethanol/water (*v*:*v* = 5:5) under solvothermal conditions at 100 °C for three days. After cooling to room temperature, the resulting *i*COF-Cl was thoroughly washed with EtOH to remove any residual reactants and was vacuum-dried overnight to obtain a brown powder. Finally, the obtained powder was soaked in acetone overnight and filtered to obtain the *i*COF-Cl. The intermediates and monomers were thoroughly characterized by using nuclear magnetic resonance (^1^H NMR and ^13^C NMR), and matrix-assisted laser desorption/ionization time-of-flight (MALDI-TOF) mass spectroscopy (Figures S1–S6).

The as-prepared *i*COF-Cl was verified through Fourier transform infrared spectroscopy (FT-IR), Raman spectroscopy, and X-ray photoelectron spectroscopy (XPS). In Figure 1b, the peak at 1332 cm^−1^ in TAPB is attributed to the stretching vibration of the C-N bond of aniline, while the characteristic signal of the nitro group appears at 1541 cm^−1^. However, these two peaks disappeared in the *i*COF-Cl. The absorption bands at 832, 1606, and 1628 cm^−1^ in the FT-IR spectrum of the *i*COF-Cl can be ascribed to the C-H out-of-plane bending vibration of aromatic group, the C=N stretching vibration of pyridinium salt, and the C=C stretching vibration of the aromatic group, respectively, suggesting the successful coupling of TAPB and Zincke-ThBiPy. The chemical state of the elemental components in the *i*COF-Cl was further characterized by XPS analysis (Table S1). As illustrated in Figure S7, both the *i*COF-Cl and Zincke-ThBiPy XPS survey spectra show distinct peaks corresponding to the elements of carbon (C), nitrogen (N), chlorine (Cl), and sulfur (S), verifying the presence of these elements in the materials. In the high-resolution S 2p spectrum, Zincke-ThBiPy reveals two predominant peaks at around 163.8 and 165.0 eV, attributed to the binding energies of the S 2p_3/2_ and S 2p_1/2_ states in the thieno[3,2-b] thiophene, respectively (Figure S7c). Compared with that of Zincke-ThBiPy, the S 2p spectrum of the *i*COF-Cl exhibits a shift towards lower binding energy, suggesting the electron donating effect of 1,3,5-triphenylbenzene [32]. Additionally, both Zincke-ThBiPy and the *i*COF-Cl exhibit similar spectrum profiles in the high resolution of C 1s and Cl 2p [33]. In contrast, the N 1s XPS spectra of the *i*COF-Cl and Zincke-ThBiPy highlight key differences in nitrogen chemical environments that stem from their unique synthesis processes and structural frameworks. For the *i*COF-Cl, the N 1s spectrum shows three primary peaks at 398 eV, 400 eV, and 402 eV, corresponding to sp^2^-hybridized nitrogen (C–N), quaternary or pyridinium nitrogen (C–N^+^), and partially reduced nitro groups (NO_2_). In contrast, Zincke-ThBiPy exhibits similar peaks for C–N at 398 eV and C–N^+^ at 400 eV, but a distinct feature emerges at 406 eV, attributed to fully oxidized nitro groups (NO_2_), indicating monomers [34]. These shifts in binding energy indicate changes in the chemical environment and electron density of the atoms, showing their incorporation into a more complex covalent framework [35]. The XPS data confirm the successful formation of the *i*COF-Cl with new interactions and coordination environments.

**Figure 2:**
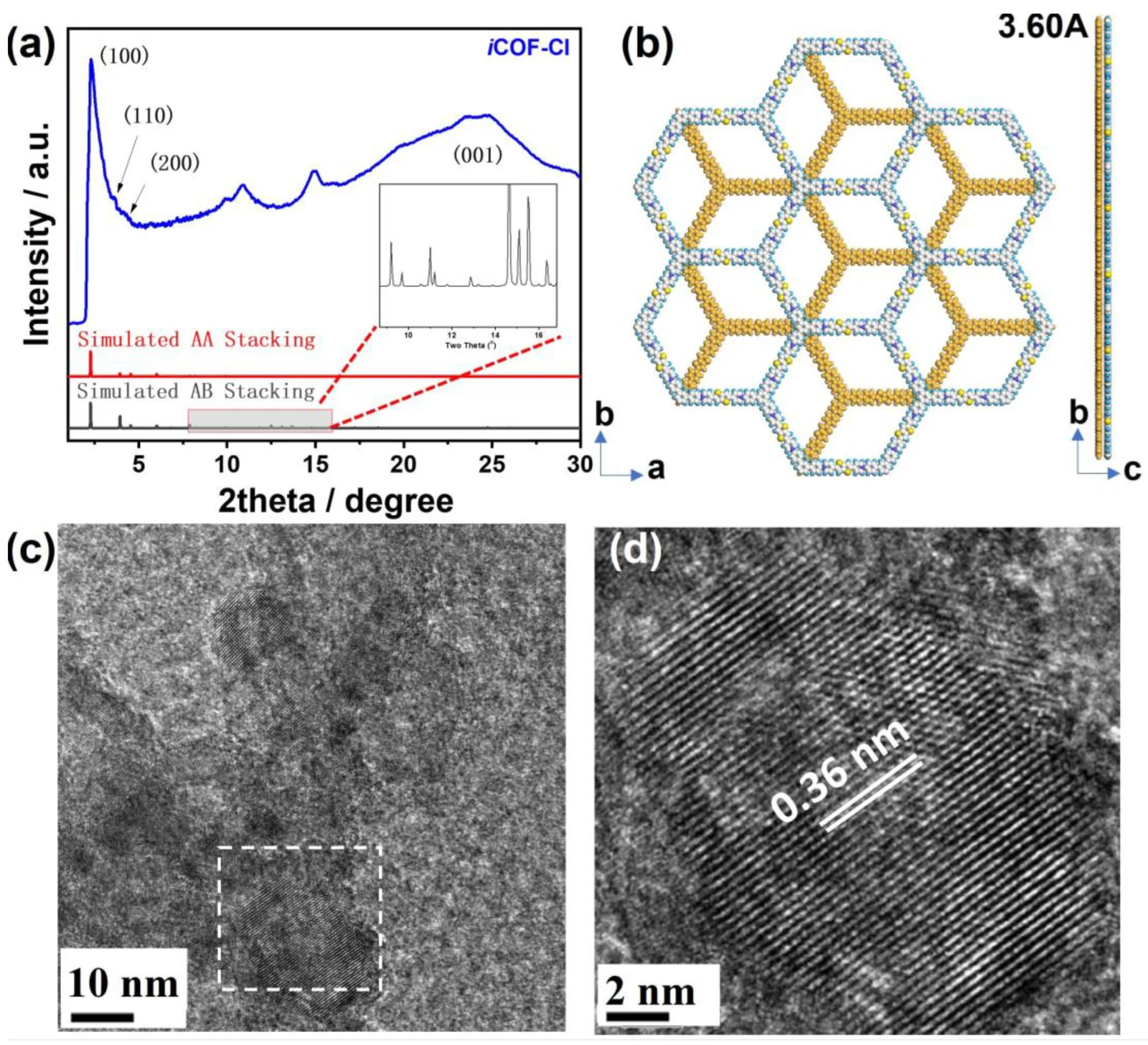
(**a**) Experimental WAXS pattern of *i*COF-Cl and calculated PXRD patterns for AA-stacking and AB-stacking. (**b**) A fragment of the layered structure shown for AB-stacked structure. (**c**) High-resolution TEM image of *i*COF-Cl, the area of white breakpoint box shows its crystalline region for enlargement analysis. (**d**) Lattice fringe for *i*COF-Cl.

The Wide-Angle X-ray Scattering (WAXS) pattern of the *i*COF-Cl reveals distinct peaks at 2*θ* = 2.2°, 3.6°, and 4.2°, corresponding to the (100), (110), and (200) facets, respectively (Figure 2a), which differ significantly from the XRD patterns of the monomer precursors (Figure S8). A broad signal at 2*θ* = 25.1° corresponds to the (001) facet, indicating an interlayer distance of 3.6 Å, which is characteristic of structures formed through π–π stacking interactions between the aromatic layers. The structural simulations of the *i*COF-Cl in the tetragonal system in AB-stacking mode yielded PXRD patterns that align well with the experimental data, confirming the AB-stacking configuration as the predominant arrangement. Notably, the experimental peaks around 2*θ* = 10.8° and 14.2° also match the simulated AB-stacking pattern, further validating this structural model. In contrast, the simulated patterns in AA-stacking mode show significant deviations in peak positions and intensities, ruling out this configuration. The combined analysis of WAXS and PXRD patterns confirms the AB-stacked tetragonal structure of the *i*COF-Cl, characterized by a well-defined interlayer spacing of 3.6 Å and distinct stacking interactions. These structural features are critical to understanding the material’s crystalline framework and potential applications. To elucidate the morphological attributes and structural composition of the prepared *i*COF-Cl material, scanning electron microscopy (SEM) was employed. Figure S9 reveals an asymmetrical bulk morphology with a size of over 1 micrometer. Transmission electron microscopy (TEM) provides direct visual evidence of the crystalline structure (Figure 2c and Figure S10). In Figure 2d, the lattice fringes with a spacing of 0.36 nm confirm the ordered arrangement, corresponding to the reflections (001) observed in the XRD pattern. This consistency between the 0.36 nm d-spacing in both XRD and high-resolution TEM data indicates that the periodic layers detected in XRD match the electron diffraction periodicity. These results collectively reinforce the conclusion that the *i*COF-Cl has a well-defined crystalline structure with π–π stacking interactions between the aromatic layers.

**Figure 3:**
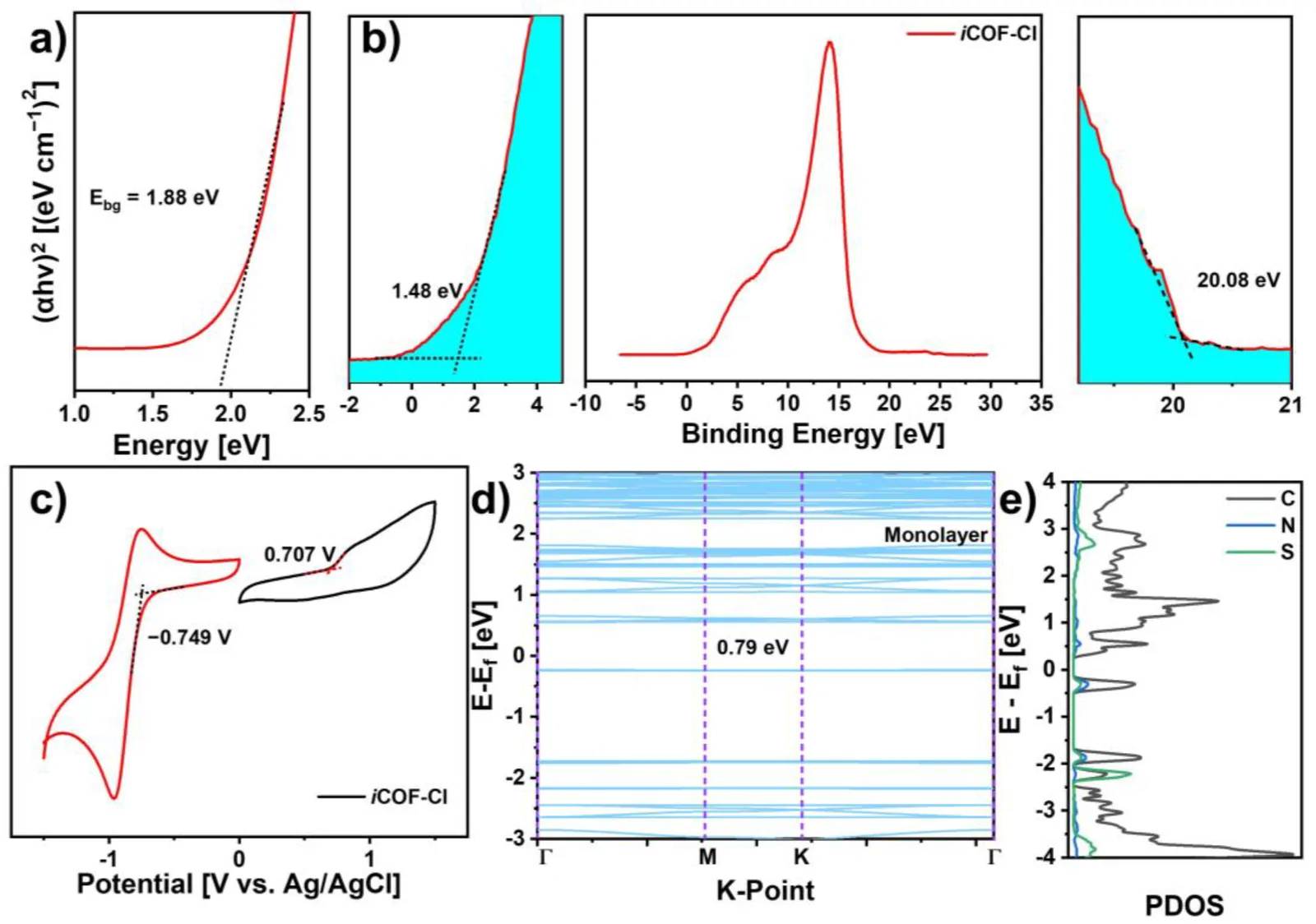
Optoelectronic properties of *i*COF-Cl. (**a**) T_auc_ plot for *i*COF-Cl. (**b**) UPS spectrum of *i*COF-Cl. The edges of the UPS spectrum are given by the intersections of two dashed lines of the tangents and the baseline, from which the UPS width is determined. (**c**) CV curves of *i*COF-Cl (calculated from corresponding onsets of redox waves referred to Fc/Fc^+^ set as −4.8 eV vs. vacuum). (**d**) Calculated band structure of *i*COF-Cl for monolayer. (**e**) Projected density of states (PDOS) of *i*COF-Cl.

The optical properties of the powder *i*COF-Cl were evaluated by using UV–vis measurements. The absorption peak around 302 nm is ascribed to the π–π transition, while the signal at 462 nm is attributed to the n–π transition of Py^+^-ThBiPy, due to the intramolecular charge transfer from the donor of ThBiPy to the acceptor of Py^+^ (Figure S11). The broad absorption region from 300 to 1000 nm is attributed to the strong intermolecular interactions in the solid state of the *i*COF-Cl. The optical bandgap (E_bg_) of the *i*COF-Cl was calculated to be 1.88 eV via the T_auc_ plot derived from the UV–vis spectrum (Figure 3a). The electronic structure of *i*COF-Cl was further characterized by ultraviolet photoelectron spectroscopy (UPS) (Figure 3b). By subtracting the measured UPS width from the excitation energy (HeI, 21.22 eV), the valence band (E_vb_) of the *i*COF-Cl was calculated to be −4.66 eV. Subsequently, the conduction band (E_cb_) was calculated to be −2.78 eV by using the equation E_cb_ = E_bg_ + E_vb_ [36]. The electronic structure of the *i*COF-Cl was further evaluated by cyclic voltammetry (CV) measurements in nitrogen-saturated acetonitrile (Figure S12). The *i*COF-Cl exhibits an irreversible oxidation peak at 0.707 eV and a one-electron reversible reduction peak at −0.749 eV. Based on the onset reduction potentials, the LUMO energy level of the *i*COF-Cl was determined to be −5.06 eV. Its HOMO energy level was calculated to be −3.18 eV with the equation HOMO = LUMO + E_bg_. These electrochemical energy levels are similar to those of the optical measurements.

To gain deep insights into its electronic properties, the band structure and partial density of states (PDOS) of the *i*COF-Cl were calculated by the DFT method (Figure 3d,e). The top of the conduction band and the bottom of the valence band in the monolayer are both flat, suggesting the effective mass of the charge carriers injected into these bands, demonstrating the semiconducting nature of the *i*COF-Cl. The band structure analysis for the monolayer reveals a band gap of 0.79 eV. Regarding the effect of AB-stacking, the band gaps of the *i*COF-Cl were narrowed to 0.60 eV, owing to the partial interlayer π−π interaction (Figure S13) [37]. The PDOS plot also demonstrates that C and S atoms contributed to the valence band, suggesting that the thieno[3,2-b] thiophene units contributed to the valence band, while the C atoms predominantly contributed to the conduction band, indicating that the 2p orbitals of the carbon atoms play a significant role in determining the electronic properties. This detailed understanding of the band structure and PDOS highlights the potential of the *i*COF-Cl for various electronic and optoelectronic applications.

The ionic conductivity of *i*COF-Cl as the pellet samples was evaluated by using a 0.5 M 1-butyl-3-methylimidazolium tetrafluoroborate (BMIMBF_4_) electrolyte through electrochemical impedance spectroscopy (EIS) measurements (Figure S14). The Nyquist plots from EIS reveal sharp semicircular arcs, indicative of ionic resistance within the material. With the increase in temperature, the radius of the semicircle decreases, suggesting enhanced ionic mobility at higher temperatures. In Figure S14b, the Arrhenius plot of log conductivity (log [S cm^−1^]) versus the reciprocal of temperature (1000/T) demonstrates the linear relationship between temperature and ion conductivity, confirming its thermally activated ionic conduction ability. The highest ionic conductivity could reach 10^−2.672^ S cm^−1^ at 333 K (60 °C). Additionally, the effect of relative humidity (RH) on the ionic conductivity of the *i*COF-Cl was also evaluated. The ionic conductivity of the *i*COF-Cl increases with the increase in RH. The ionic conductivity of the *i*COF-Cl is 5.02 × 10^−3^ S cm^−1^ at 57% RH, and it can reach 1.219 × 10^−2^ S cm^−1^ at 98% RH (Figure S14c and Tables S2 and S3). This phenomenon is attributed to the formation of hydrogen bonds by the adsorption of water molecules, facilitating the enhanced mobility of charge carriers within the material [38]. These findings underscore the potential of the *i*COF-Cl as a highly conductive material under varying environmental conditions, making it suitable for advanced electrochemical applications.

**Figure 4:**
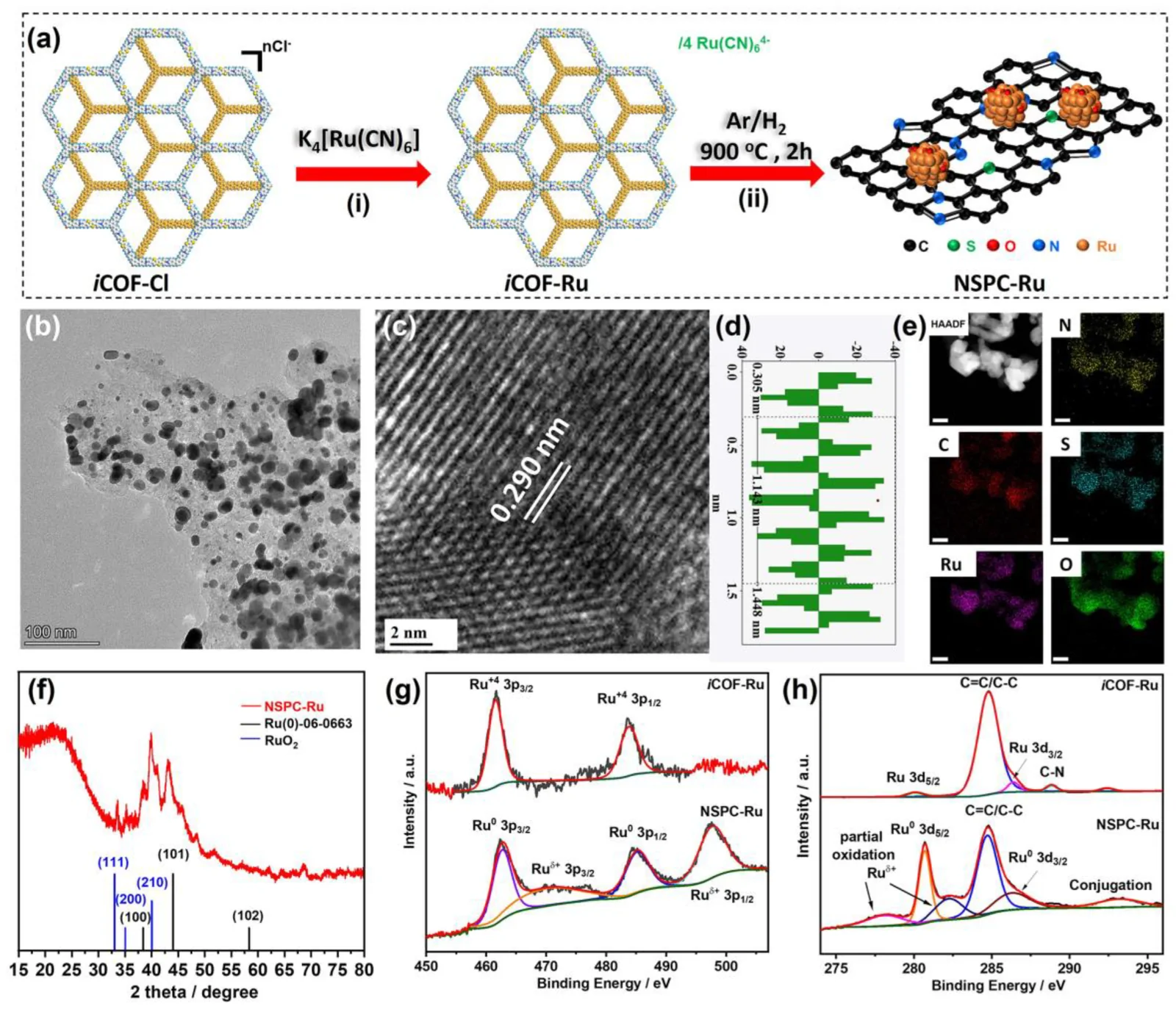
(**a**) Preparation of NSPC-Ru through anion-exchanging strategy: (i) aqueous solution of *i*COF-Cl and [K_4_(Ru(CN_6_)] mixed for 7 days at r.t.; (ii) high-temperature treatment of *i*COF-Ru at 900 °C. (**b**) HRTEM image of NSPC-Ru. (**c**) Lattice fringes for NSPC-Ru. (**d**) Line profile of lattice fringes. (**e**) EDS elemental mapping for NSPC-Ru, scale bar: 200 nm. (**f**) XRD pattern for NSPC-Ru compared with standard metallic Ru (JCPDS card No. 06-0663) and RuO_2_ (JCPDS card No. 43-1027). (**g**) XPS analysis of Ru 3p spectra of iCOF-Ru and NSPC-Ru, black color for raw data, red color for smooth data, dark green for baseline, violet color for Ru^0^ 3p_3/2_, blue for Ru^0^ 3p_1/2_, orange color for Ru^&+^ 3p_3/2_, cyan color for Ru^&+^ 3p_1/2_. (**h**) XPS analysis of C 1s and Ru 3d spectra of iCOF-Ru and NSPC-Ru, black color for raw data, red color for smooth data, dark green for baseline, blue for C=C/C-C, cyan for C-N, light green for Ru4+ 3d5/2, magenta for Ru4+ 3d_3/2_, violet color for Ru^0^ 3d_5/2_, wine color for Ru^0^ 3d_3/2_, orange for Ru^&+^.

Owing to its charge conjugated framework and high residual carbon yield, this *i*COF-Cl could be applied as the precursor for the construction of metal/porous carbon composites [39,40]. As illustrated in Figure 4a, the chloride anions (Cl^−^) in the *i*COF-Cl are replaced by hexacyanoruthenate anions ([Ru(CN)_6_]^4−^) by a typical anion-exchange procedure, resulting in the construction of the *i*COF-Ru with the incorporation of Ru species within the COF. The High-Angle Annular Dark-Field Scanning Transmission Electron Microscopy (HAADF-STEM) image clearly shows the white spots corresponding to the ruthenium (Ru) element due to its higher atomic number (Figure S15), evidencing the presence of Ru species within the matrix of the COF. The uniform distribution of Ru species in the *i*COF-Ru was confirmed by Energy-Dispersive X-ray Spectroscopy (EDS) elemental mapping. Owing to its high residual carbon yield (Figure S16), the *i*COF-Ru was subjected to pyrolysis at 900 °C for 2 h. This high-temperature treatment reduces the ruthenium anions to ruthenium nanoparticles to yield a N/S-co-doped porous carbon material embedded with Ru/RuO_2_ nanoparticles (NSPC-Ru). The microstructure of the prepared NSPC-Ru was investigated by using TEM, as shown in Figure 4b. The TEM image reveals that the as-prepared NSPC-Ru nanosheets exhibit a uniform hexagonal morphology (Figure 4c), and the HRTEM image displays lattice fringes with a d-spacing of 0.290 nm for metallic Ru^0^ (Figure 4d and Figure S17). Figure 4e depicts the EDS mapping of the NSPC-Ru, revealing the uniform distribution of C, N, Ru, and S throughout the structure. The XRD pattern of the NSPC-Ru (Figure 4f) shows prominent peaks at 2*θ* = 38.6°, 44.1°, and 58.4°, which correspond to the characteristic peaks of Ru^0^ metal (JCPDS card No. 06-0663), while these peaks at 2*θ* = 32.1°, 35.1°, and 40.0° are attributed to the characteristic peaks of RuO_2_ (JCPDS card No. 43-1027). These analyses confirm the successful synthesis of the NSPC-Ru with Ru/RuO_2_ nanoparticles [41]. The valence states of N, C, S, and Ru elements in the *i*COF-Ru and NSPC-Ru were further evaluated by XPS measurements (Figure S18). For the *i*COF-Ru, the high-resolution N 1s XPS spectrum reveals the presence of multiple nitrogen environments, indicating variations in chemical bonding. The deconvoluted peaks show contributions from C–N at 398.7 eV, C–N^+^ at 400.3 eV, quaternary nitrogen at 401.2 eV, and reduced NO_2_ at 403.5 eV. In contrast, the NSPC-Ru exhibits distinct nitrogen species, including pyridinic nitrogen, pyrrolic nitrogen, and quaternary nitrogen, at 398.5 eV, 399.8 eV, and 401.1 eV, respectively (Figure S18b) [34]. These differences in nitrogen configurations suggest variations in the electronic structure and surface chemistry between the two materials, modulating electron density, adsorption properties, and interaction with reactants. In the high-resolution S 2p spectrum, the *i*COF-Ru exhibits two predominant peaks corresponding to the binding energies of S 2p_3_/_2_ at 163.8 eV and S 2p_1_/_2_ at 165.0 eV, which can be attributed to sulfur species in the thieno[3,2-b]thiophene framework (Figure S18c) [32]. In comparison, the S 2p spectrum of the NSPC-Ru displays a more pronounced sulfur peak, indicating higher sulfur content. In Figure 4g, the Ru 3p XPS spectrum of the *i*COF-Ru shows two peaks at 461.6 eV and 484 eV, corresponding to the Ru 3p_3_/_2_ and Ru 3p_1_/_2_ of the high oxidation state of Ru^4+^ species, respectively, suggesting the successful incorporation of [Ru(CN)_6_]^4−^ in the iCOF-Ru. Additionally, two Ru 3d peaks at 280.1 eV (Ru 3d_5_/_2_) and 285.8 eV (Ru 3d_3_/_2_) are also detected (Figure 4h). Moreover, the C 1s spectra reveal that the dominant peak at 284.8 eV, corresponding to C=C bonds, demonstrates the characteristic of sp^2^ hybridized carbon in the *i*COF-Ru. These results confirm that the [Ru(CN)_6_]^4−^ anions are loaded in the framework of the *i*COF without damage to the polymeric structure. For the NSPC-Ru, slight red shifts in the binding energies of both the Ru 3p and Ru 3d spectra are observed, suggesting the successful integration of Ru^0^ into the carbon framework. Additionally, the NSPC-Ru exhibits two new peaks in the Ru 3p spectra (469.8 eV and 497.4 eV) and in the Ru 3d spectra (278.0 eV and 282.3 eV), which can be attributed to surface interactions with oxygen. These peaks may also originate from interfacial interactions with carbon, possibly influenced by sulfur and nitrogen [42]. Moreover, additional peaks in Ru^4+^ 3p and Ru 3d can be ascribed to RuO_2_ in the skeleton of porous carbon [43]. These results demonstrate the successful synthesis of Ru/RuO_2_ nanoparticle-embedded N/S-co-doped porous carbon.

**Figure 5:**
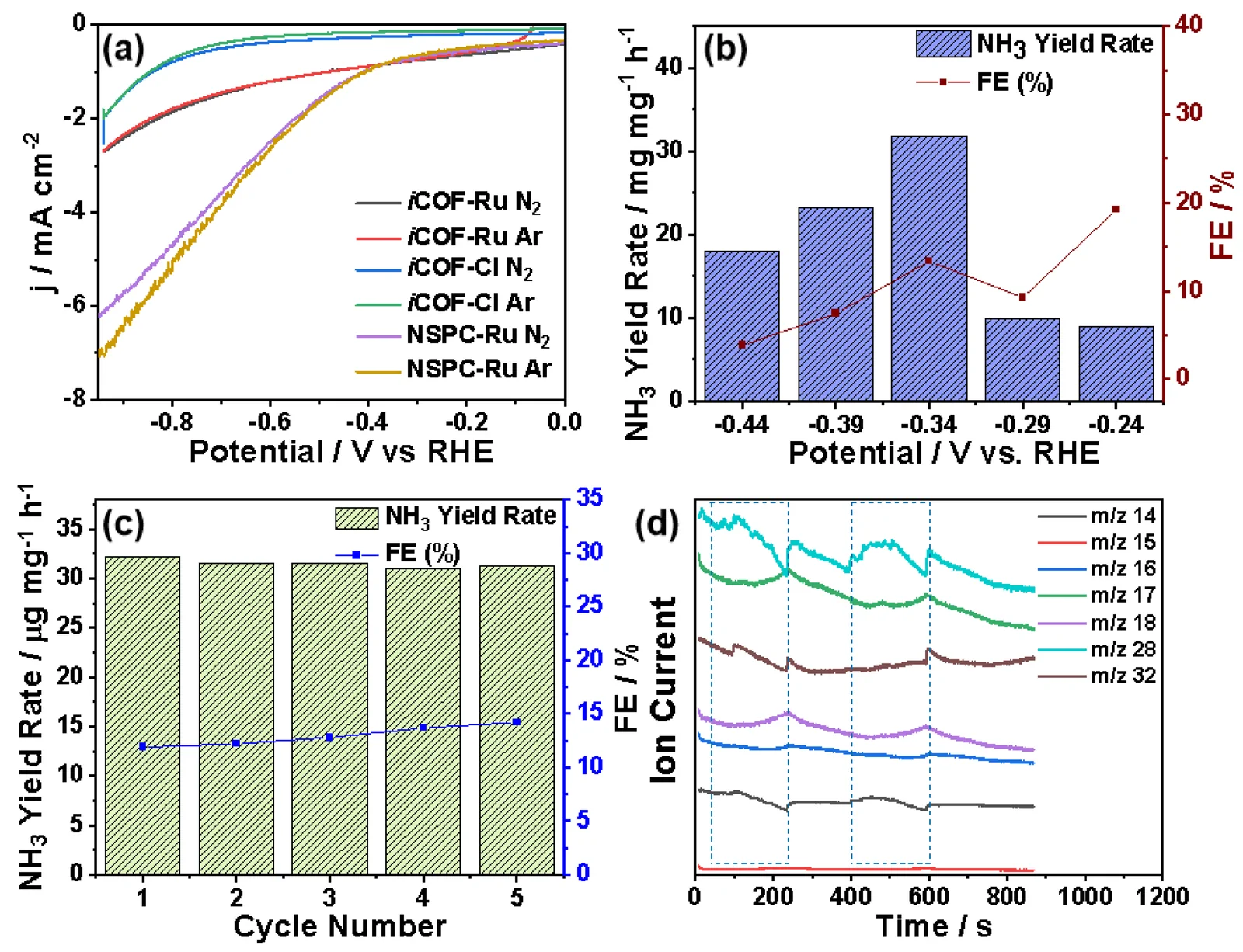
(**a**) LSV curves for *i*COF-Ru, *i*COF-Cl, and NSPC-Ru in Ar and N_2_. (**b**) FE and NH_3_ yield rates for NSPC-Ru for eNRR. (**c**) FE and NH_3_ yield rates for NSPC-Ru at potential of −0.34 V vs. RHE after five consecutive experiments. (**d**) In situ DEMS investigation: ion current responses of *m*/*z* signal in different time intervals relative to LSV curve.

Owing to the porous structure with uniform distribution of Ru/RuO_2_ nanoparticles, the electrochemical nitrogen reduction reaction (eNRR) of the NSPC-Ru test was thoroughly evaluated by using a three-electrode setup in 0.1 M potassium sulfate (K_2_SO_4_) solutions (pH = 7.0) [44]. As illustrated in Figure S19a, the NSPC-Ru catalyst was loaded onto carbon paper, serving as the working electrode, with a Nafion 117 membrane separating it from the counter electrode. All electrode potentials were measured with a commercial Ag/AgCl reference electrode and standardized to the reversible hydrogen electrode (RHE) scale. Linear sweep voltammetry was performed in the Ar- and N_2_-saturated electrolytes comprising the samples, and the current density was increased with the increase in the negative potential. Compared with that under the Ar-saturated condition, the current density under the N_2_-saturated condition is more negative, suggesting the possible occurrence of NRRs in all samples (Figure 5a). For example, at −0.6 V vs. RHE, the current density of −2.80 mA cm^−2^ under the N_2_-saturated condition is higher than that (−2.46 mA cm^−2^) under the Ar-saturated condition. By using ammonium sulfate to establish the corresponding standard curve by UV–vis measurements, the product of NH_3_ was evaluated via the indophenols-blue method [45]. The product of the NRR is NH_3_, and N_2_H_4_ is not detectable in the electrolyte (Figures S20–S24). Figure 5b shows the NH_3_ yield rate and Faradaic efficiency (FE) of the NSPC-Ru in the applied potential range of −0.24–−0.44 V vs. RHE. At −0.34 V vs. RHE, the highest NH_3_ yield rate is 32.0 μg h^−1^ mg^−1^ with a corresponding FE of 13.2%, which is comparable to other reported works (Table S4). The cycle stability of the NSPC-Ru was carried out at −0.34 V vs. RHE. As shown in Figure 5c, the NH_3_ yield rates of the NSPC-Ru remain around 32.0 μg h^−1^ mg^−1^ over five consecutive eNRR cycles, and the values of FE have a slight incline over repeated cycles. This is further supported by Figure S21, indicating that the NSPC-Ru and the *i*COF-Ru possess a higher electrochemically active surface area (ECSA) compared with the *i*COF-Cl, as reflected by their larger C_dl_ values. A higher C_dl_ correlates with more active sites available for catalytic reactions, which is crucial to enhancing nitrogen reduction reaction (NRR) efficiency. Moreover, the NSPC-Ru demonstrated exceptional stability and durability, maintaining a steady current density over 15 h of continuous electrolysis, as seen in Figure S26b. On the other hand, the highest NH_3_ yield rate could reach 11.2 μg h^−1^ mg^−1^ for the *i*COF-Ru at −0.29 V vs. RHE, with a much lower current density under the N_2_-saturated condition, as well as the Ar-saturated condition (Figure S27). These results demonstrate that the NSPC-Ru exhibits high electrochemical durability for nitrogen reduction. To further understand the catalytic activity of the NSPC-Ru, online differential electrochemical mass spectrometry (DEMS) measurements were conducted to monitor volatile intermediates (Figure S25) [46]. In the eNRR process, the primary detected species are nitrogen (N_2_, *m*/*z* = 28) and ammonia (NH_3_, *m*/*z* = 17, 18), indicating the successful conversion of nitrogen to ammonia. Although hydrazine (N_2_H_4_), a known intermediate, could theoretically be detected at *m*/*z* = 32 with fragments at *m*/*z* = 14, 15, and 16, it is not clearly observed in the data. This is likely due to its rapid conversion to ammonia, preventing its accumulation, or because its concentration is too low compared with nitrogen and ammonia. Additionally, fragmentation overlap with species like ammonia, water, and oxygen may obscure the hydrazine signals. The catalyst efficiently converts nitrogen to ammonia, bypassing or minimizing the stabilization of hydrazine as a long-lived intermediate, resulting in the detection of only the stable products (N_2_ and NH_3_). The synergistic electronic effect of Ru/RuO_2_ enhances catalytic performance by maximizing active site exposure, improving mass transport, and facilitating efficient electron transfer for N_2_ activation. Additionally, the highly ordered ionic pore channels in *i*COF-based catalysts minimize transport resistance, ensuring a more efficient and uniform reaction environment. Unlike conventional COFs, *i*COFs incorporate charged moieties within their framework, promoting selective ion transport and optimizing catalytic efficiency. Their structural precision and electrochemical stability make them highly suitable for applications requiring sustained performance under extreme conditions. These attributes collectively establish *i*COFs as advanced catalytic materials for the nitrogen reduction reaction (NRR), where enhanced charge transport and ion mobility play a crucial role in improving reaction kinetics and selectivity [21,44].

## 4. Conclusions

The study of the thienothiophene-bridged viologen-based ionic covalent organic framework (*i*COF-Cl) and its derivative (NSPC-Ru) highlights significant advancements in material synthesis and catalytic performance. The *i*COF-Cl, synthesized by reacting tris(4-aminophenyl) benzene (TAPB) with Zincke-ThBiPy at 100 °C, was confirmed to have an AB-stacked structure with an optical band gap of 1.88 eV. The NSPC-Ru was subsequently synthesized by reducing Ru anions in the *i*COF-Cl, achieving high crystallinity and the incorporation of Ru/RuO_2_ nanoparticles. The catalytic performance of the NSPC-Ru was evaluated for the electrocatalytic conversion of N_2_ to NH_3_ under ambient conditions. Experimental results demonstrated an NH_3_ yield rate of 32.0 μg mg^−1^ h^−1^ and a Faradaic efficiency of 13.2% at −0.34 V vs. RHE, with notable stability over repeated cycles. Moreover, the catalyst exhibited remarkable stability and durability, sustaining a steady current density over 15 h of continuous electrolysis. This study establishes a robust platform for the further exploration and development of metal-embedded heteroatom-doped porous carbon sheets and the potential of the NSPC-Ru as an exceptionally efficient electrocatalyst for advanced electrocatalytic systems.

## Data Availability

The data presented in this study are available upon request from the corresponding author.

## Supplementary Materials

The following supporting information can be downloaded at: https://www.mdpi.com/article/10.3390/polym17040543/s1, Figures S1–S5: ^1^H NMR and ^13^C NMR, Figure S6: MALDI-TOF, Figure S7: XPS analysis, Figure S8: XRD spectra, Figures S9 and S10: SEM and TEM images, Figure S11: UV–vis spectrum, Figure S12: CV analysis, Figure S13: Calculated band structure, Figure S14: Nyquist plots, Figure S15: HAADF-STEM, Figure S16: TGA analysis, Figure S17: HRTEM analysis, Figure S18: XPS analysis, Figures S19–S24: eNNR tests, Figure S25: online DEMS analysis, Figure S26: (a) LSV curves of NSPC-Ru before and after long-term stability test. (b) Long-term stability of NSPC-Ru. Figure S27: (a) Chronoamperometric curves for NSPC-Ru after every one hour at 5 different voltages. (b) UV–vis absorption spectra. (c) NH3 yield rates for iCOF-Ru for eNRR. Table S1: XPS analysis, Table S2: Conductivity results, Table S3: Comparison performance of conductivity, Table S4. Comparison of different catalysts in electrochemical nitrogen reduction reaction.
