# Supplementary material for: Anion-Exchange Strategy for Ru/RuO2-Embedded N/S-Co-Doped Porous Carbon Composites for Electrochemical Nitrogen Fixation
Source: Polymers (Basel). 2025 Feb 19;17(4):543. doi: 10.3390/polym17040543 (PMC11858955; doi:10.3390/polym17040543)
Supplement: Supplementary file 1 [file polymers-17-00543-s001.zip › polymers-3473010-supplementary.pdf]

Supplementary Materials

Anion-Exchange Strategy for Ru/RuO_2-Embedded N/S *co*-doped Porous Carbon Composites for Electrochemical Nitrogen Fixation

Shahzeb Ali Samad [1], *Xuanzi Ye* [1], *Zhiya Han* [2], *Senhe Huang* [1], *Chenbao Lu* [1], *Junbo Hou* [3], *Min Yang* [2], *Zhenyu Zhang* [4,*], *Feng Qiu* [5,*], *and Xiaodong Zhuang* [1,6,*]

[1] The Soft2D Lab, State Key Laboratory of Metal Matrix Composites, Shanghai Key Laboratory of Electrical Insulation and Thermal Ageing, School of Chemistry and Chemical Engineering, Shanghai Jiao Tong University, 800 Dongchuan Road, Shanghai 200240, China

[2] School of Materials, Shanghai Dianji University, 300 Shuihua Road, Pudong New Area District, Shanghai 201306, China

[3] Power System Resources Environmental Technology Co. Ltd, 585 Changan North Road, Haiyan 314399, China

[4] Shanghai Nuclear Engineering Research and Design Institute Co., Ltd., 169 Tianlin Road, Xuhui District, Shanghai 200030, China

[5] School of Chemical and Environmental Engineering, Shanghai Institute of Technology, 100 Haiquan Road, Shanghai 201418, China.

[6] Frontiers Science Center for Transformative Molecules, Zhang Jiang Institute for Advanced Study, Shanghai Jiao Tong University, 429 Zhangheng Road, Shanghai 201203, China

Correspondence: zhangzhenyu@snerdi.com.cn; fengqiu@sit.edu.cn; zhuang@sjtu.edu.cn

Table of Contents

Section A. Electrochemical measurements for CV 1

Section B. Cathode preparation for NRR.. 1

Section C. Electrochemical measurements for NRR.. 1

Section D. Determination of NH_4^+ via an indophenols-blue method.................... 2

Calculation of NH_3 yield rate 2

Calculation of FE... 3

Section E. Supporting figures and tables.. 3

Section A. Electrochemical measurements for CV

Cyclic voltammetry (CV) was conducted by using a CHI 650E electrochemical analyzer in dry ACN with purified tetrabutylammonium hexafluorophosphate ($TBAPF_6$, 0.1 M) as the electrolyte at 298 K. A standard three-electrode setup was utilized, consisting of a glassy carbon working electrode (0.3 M surface area) and a platinum wire as the counter electrode. Prior to use, the glassy carbon working electrode was polished by using alumina suspension and rinsed with acetone. Potential measurements were referenced to the Ag/AgCl electrode. The specimen was transferred in a wet state onto the glassy carbon working electrode surface and allowed to dry at room temperature for 30 minutes. The calibration of the CV curves was performed by using the ferrocene/ferrocenium (Fc/Fc^+) redox couple as an external standard, which was measured under identical conditions prior to and following sample measurement. The energy level of Fc/Fc^+ was determined to be -4.8 eV in relation to vacuum. The half-wave potential of the Fc/Fc^+ redox couple ($E_{1/2(Ferrocene)}$) was estimated with

$$E_{1/2(Ferrocene)} = (E_{ap} + E_{cp}) / 2 \qquad \text{Equation (1)}$$

where E_{ap} and E_{cp} are the anodic and cathodic peak potentials, respectively.

The HOMO and the LUMO energy levels were calculated with equations (2) and (3), respectively, as follows:

$$E_{HOMO}^{CV} = [(E_{ox} - E_{1/2(Ferrocene)}) + 4.8]\ eV \qquad \text{Equation (2)}$$

$$E_{LUMO}^{CV} = [(E_{red} - E_{1/2(Ferrocene)}) + 4.8]\ eV \qquad \text{Equation (3)}$$

where E_{ox} and E_{red} are the onset oxidation and reduction potentials relative to the Ag/Ag^+ reference electrode.

Section B. Cathode preparation for NRR

Typically, a homogeneous catalyst ink with 3 mg of catalyst and 300 μL of Nafion solution (5 wt%) was dispersed by sonication for 2 hours. Then, 100 μL of the above ink was loaded onto a carbon paper electrode with an area of 1 × 1 cm^2 (CP, 1 × 2.5 cm^2) and dried in a glove box filled with N_2 atmosphere at room temperature.

Section C. Electrochemical measurements for NRR

The electrochemical experiments were carried out in an H-type cell at room temperature. The reduction of N_2 (99.999%) was performed in a gas-tight, two-compartment cell which was separated by a Nafion 117 membrane. In a typical test, the catalyst was deposited on carbon paper as the working electrode, the Ag/AgCl electrode was used as the reference electrode, and

the Pt wire was used as the counter electrode. Before the experiment, the Nafion 117 membrane was boiled in ultrapure water for 1 hour, treated in H_2O_2 (5%) aqueous solution at 80°C for 1 hour, soaked in 0.5 M H_2SO_4 for 2 hours at 80°C, and subsequently boiled in water for 6 hours. Electrochemical measurements were operated on a Bio-Logic multichannel electrochemical workstation (VSP-300).

Before the experiment, the electrolyte in the cathode cell was bubbled with N_2 for 30 min to ensure that the air was excluded in the electrolyte. The electrolyte of the first cycle (20 CV scans and 1 hour electrolysis) was discarded to exclude any possible pre-absorbed ammonia contamination for each newly prepared electrode. Each electrolytic process was sustained for 1 hour at each potential. All potentials were measured against an Ag/AgCl reference electrode and converted to the RHE reference scale by using the equation E (vs. RHE) = E (vs. Ag/AgCl) + 0.197 V + 0.0591 × pH. Linear sweep voltammetry (LSV) was carried out in a voltage window from -1.1 V to 0 V vs. RHE with a scan rate of 20 mV s^{-1}. For eNRR experiments, the test was conducted in N_2-saturated 0.1 M K_2SO_4 (30 mL) for 1 hour at each potential. The electrochemically active surface areas (ECSAs) were estimated by measuring the capacitive current associated with double-layer charging from the scan-rate dependence of cyclic voltammetry (CV). The potential window of CV was from -0.65 V to -0.75 V vs. RHE with scan rates of 10, 20, 30, 40, and 50 mV s^{-1}.

Section D. Determination of NH_4^+ via an indophenols-blue method

In detail, 2 mL of 1 M NaOH solution containing salicylic acid (5 wt%) and sodium citrate (5 wt%) was added into 2 mL of the electrolyte after N_2 electroreduction, followed by the addition of 1 mL of 0.05 M NaClO and 0.2 mL of $C_5FeN_6Na_2O{\cdot}2H_2O$ (1 wt%). After shaking and standing for 1 h, the concentration of NH_4^+ was measured by using a UV–vis spectrophotometer in a wavelength range from 500 nm to 800 nm. NH_4^+ show typical absorption of ultraviolet light at the wavelength of 655 nm, in which the absorption value is in proportion to the concentration of NH_4^+. The standard concentration–absorption curve was calibrated by using standard $(NH_4)_2SO_4$ solution with a series of concentrations (0, 0.5, 1.0, 2.0, 5.0, 10.0, and 20.0 μg mL^{-1}).

Calculation of NH_3 yield rate

The NH_3 yield rate was calculated via the following equation:

$$R_{NH_3} = \frac{CV}{MT} \qquad \text{Equation (4)}$$

where the following apply:

R_{NH3}: the NH_3 yield rate;

C: the measured mass concentration of NH_4^+;

V: the volume of the electrolyte (30 mL, in this case);

M: the mass of the catalyst (1 mg, in this case).

Calculation of FE

The NH_3 yield rate was calculated via the following equation:

$$FE = CVNF/(QM) \qquad \text{Equation (5)}$$

where the following apply:

C: the measured mass concentration of NH_4^+;

N: the number of electrons transferred for product formation, which is 3 for NH_3;

F: the Faraday constant, 96485 C mol^{-1};

M: the relative molecular mass of NH_3, which is 18 g mol^{-1}.

Section E. Supporting figures and tables

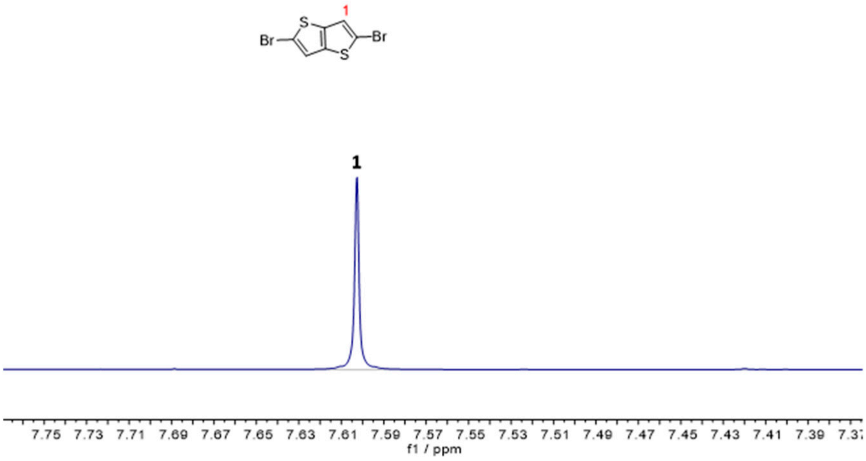

Figure S1. ^1H NMR of Th-Br.

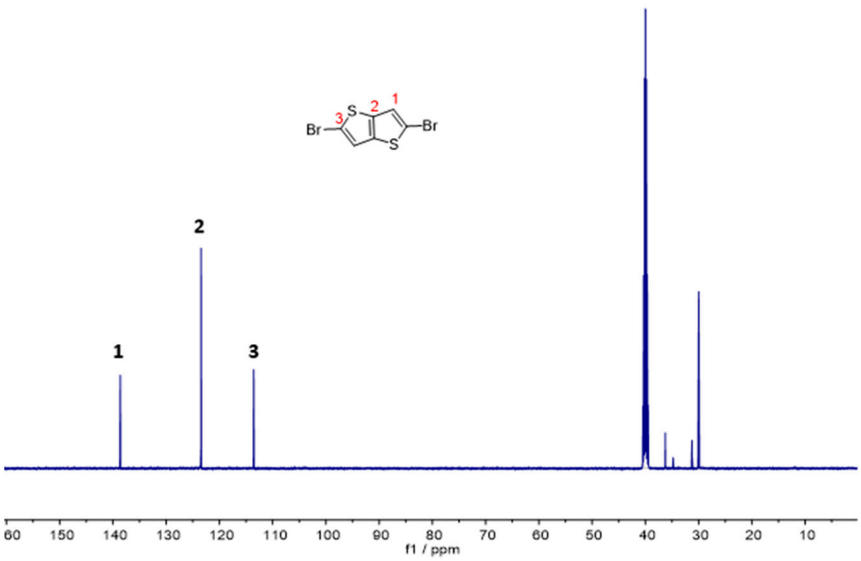

Figure S2. ^{13}C NMR of Th-Br.

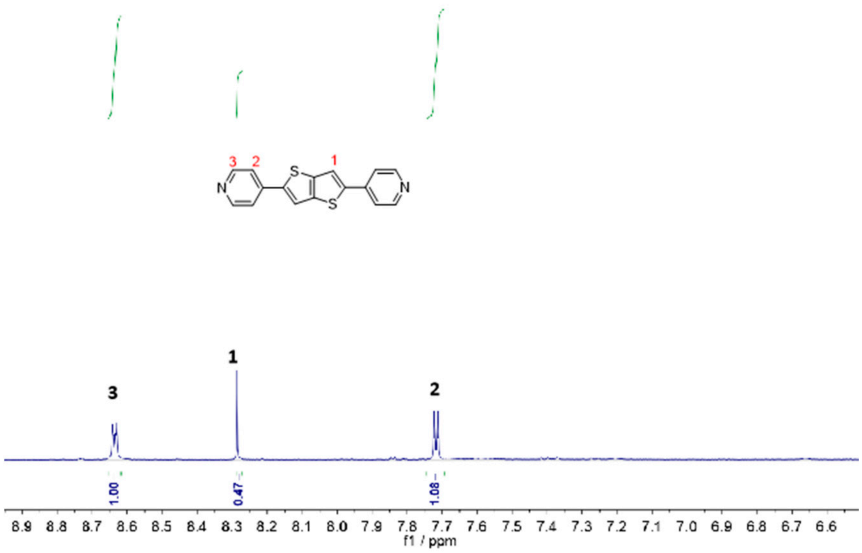

Figure S3. ^{1}H NMR of ThBiPy.

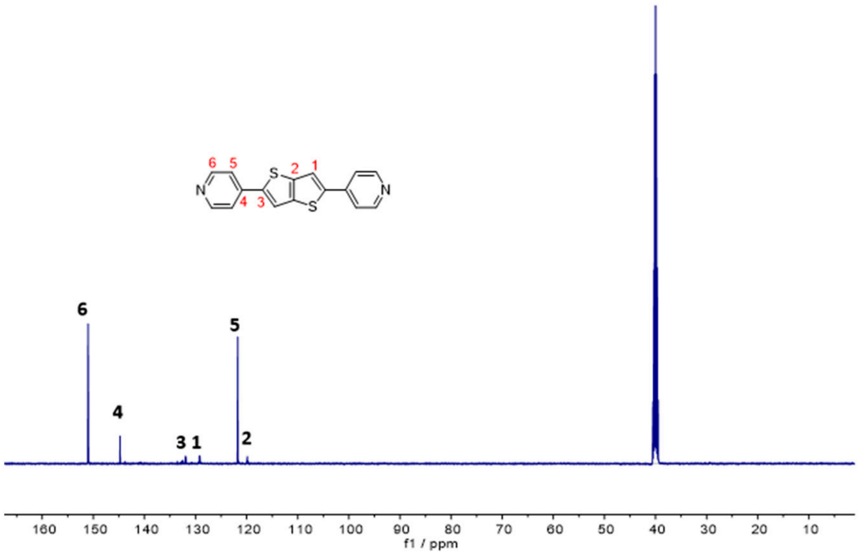

Figure S4. ^{13}C NMR of ThBiPy.

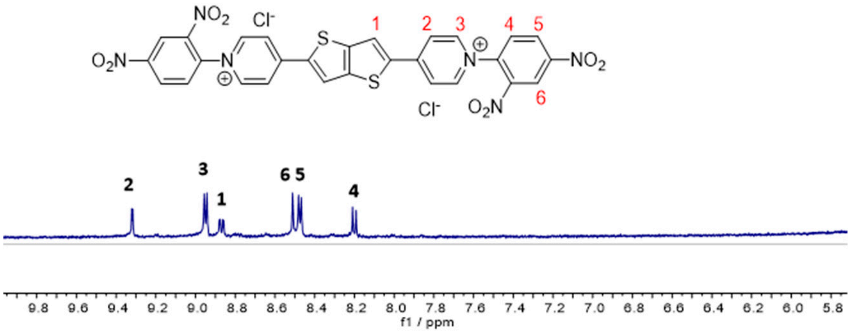

Figure S5. ^{1}H NMR of Zincke-ThBiPy.

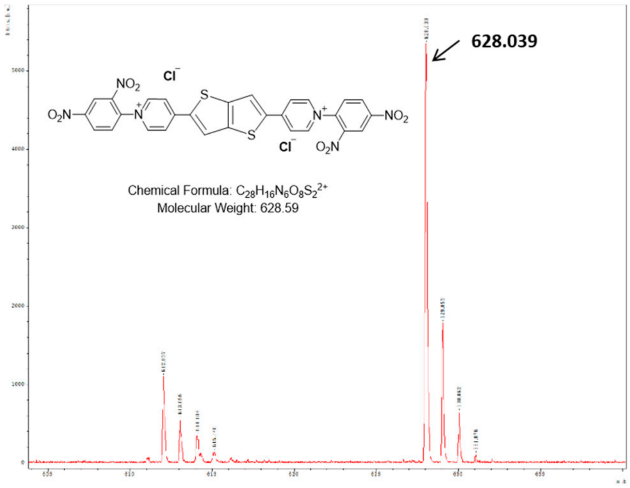

Figure S6. MALDI-TOF MS of Zincke-ThBiPy.

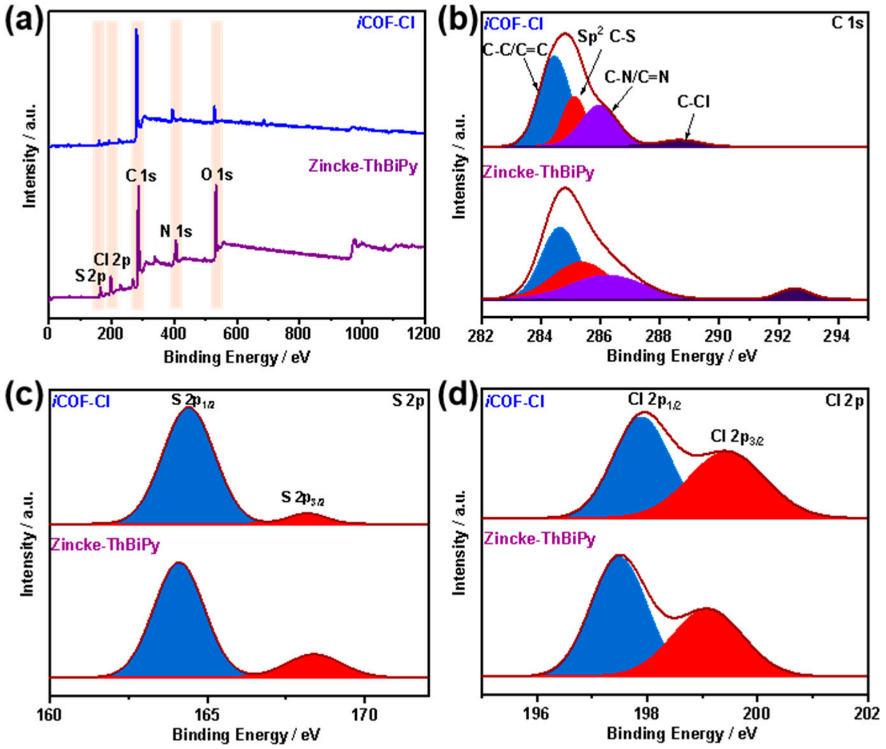

Figure S7. XPS analysis of *i*COF-Cl and Zincke-ThBiPy. a) Survey scan. b) C 1s. c) S 2p. d) Cl 2p.

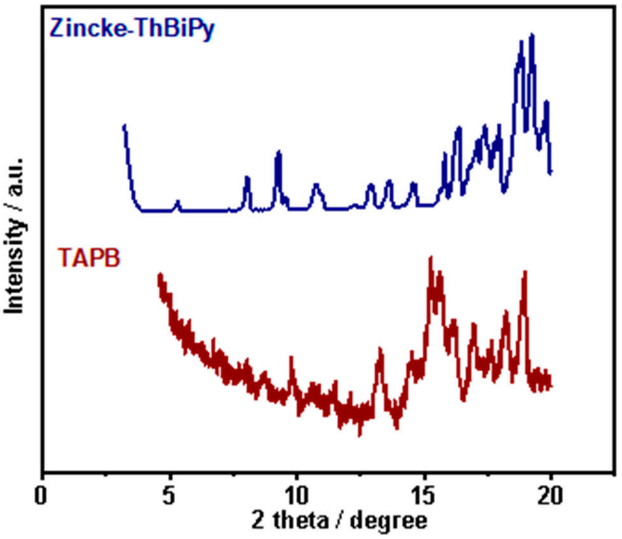

Figure S8. XRD spectra of Zincke-ThBiPy and TAPB.

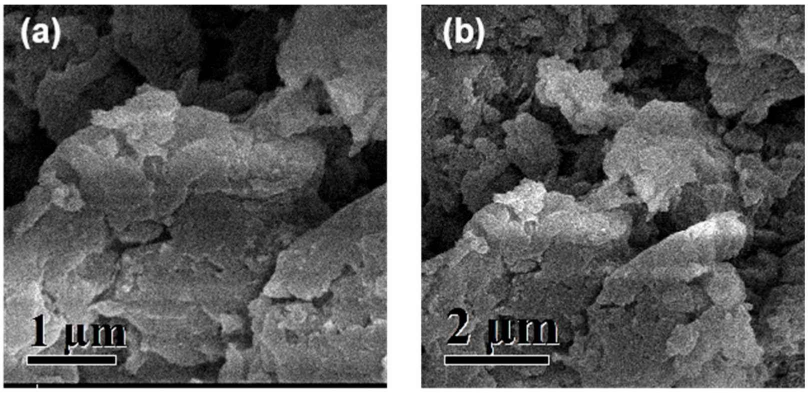

Figure S9. SEM analysis of *i*COF-Cl.

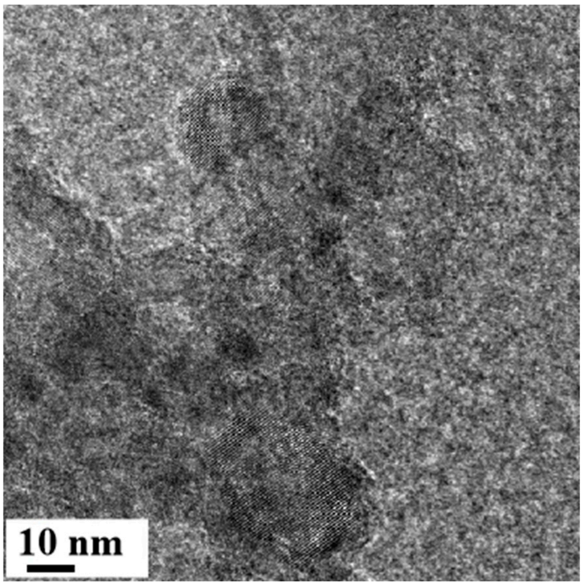

Figure S10. HRTEM analysis of *i*COF-Cl.

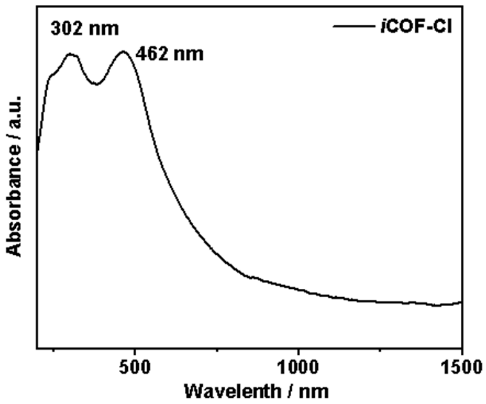

Figure S11. UV–vis spectrum of *i*COF-Cl.

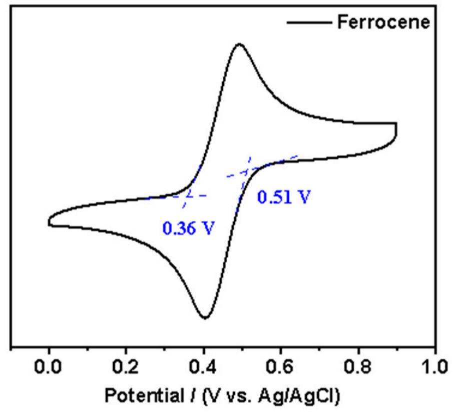

Figure S12. CV analysis of Fc/Fc+ for *i*COF-Cl.

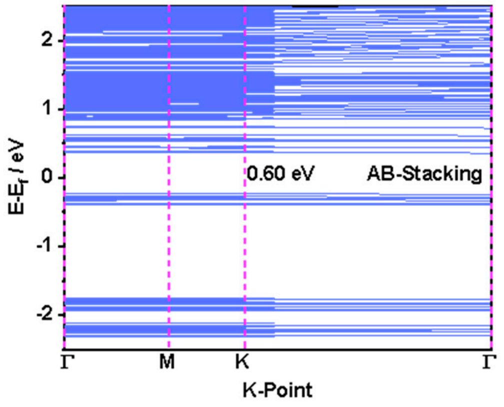

Figure S13. Calculated band structure of *i*COF-Cl for AB-stacking.

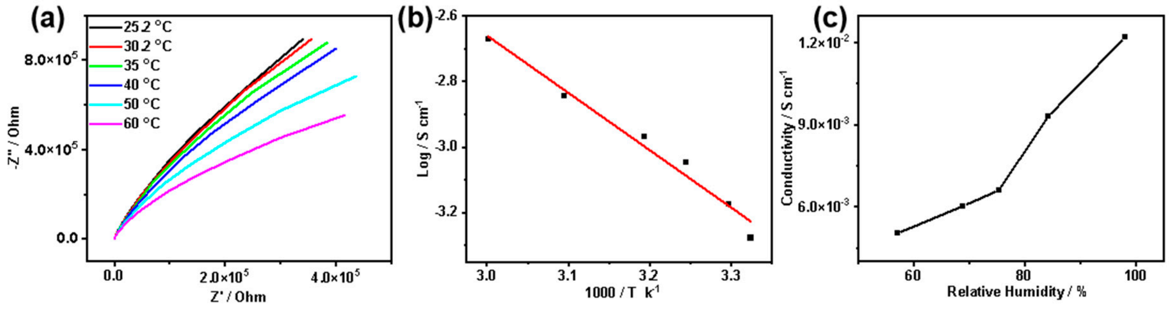

Figure S14. a) Nyquist plots of *i*COF-Cl. b) Arrhenius plots of ionic conductivity of *i*COF-Cl. c) Ionic conductivity of *i*COF-Cl at different relative humidity values.

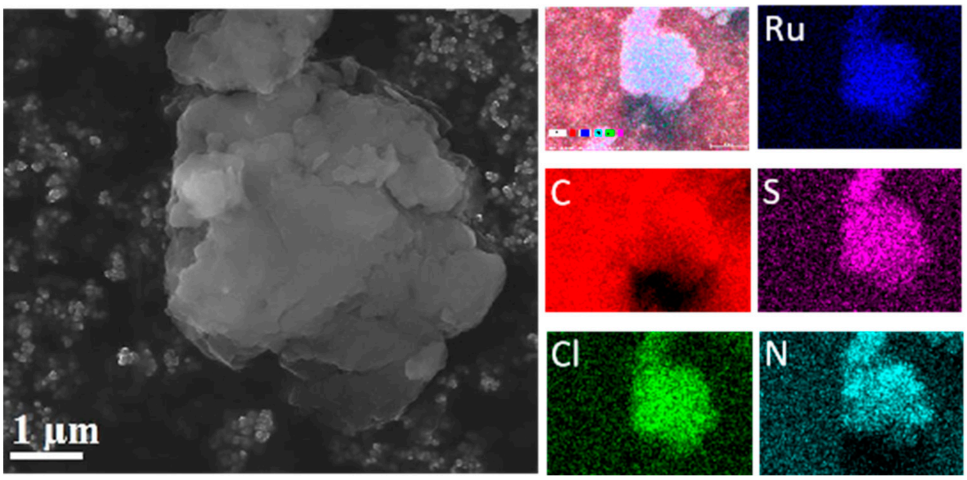

Figure S15. HAADF-STEM image and EDS elemental mapping images of *i*COF-Ru.

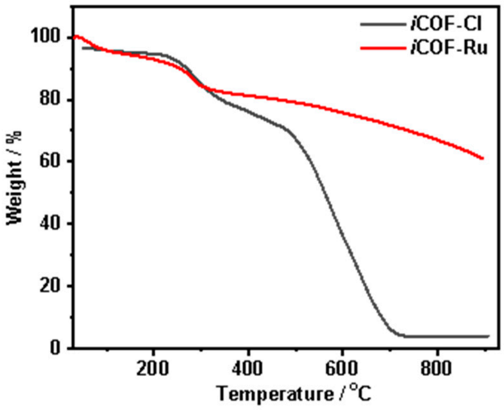

Figure S16. TGA analysis of *i*COF-Cl and *i*COF-Ru.

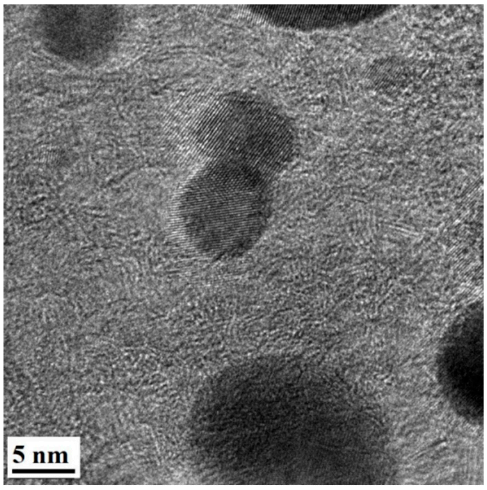

Figure S17. HRTEM analysis of *i*COF-Ru.

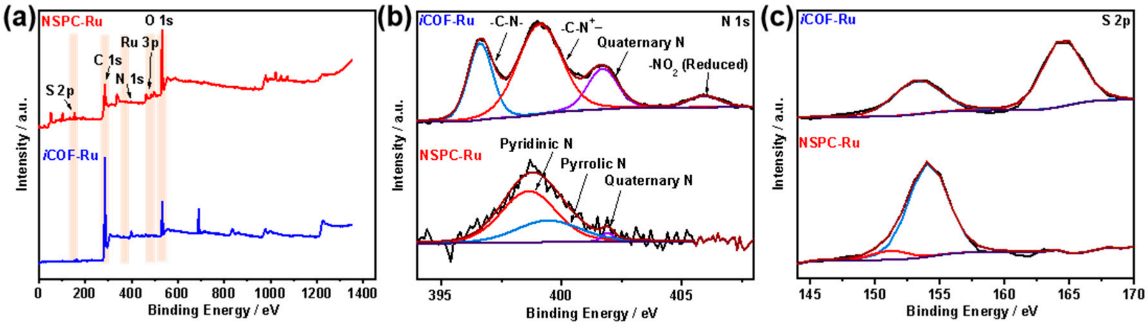

Figure S18. XPS analysis of NSPC-Ru and *i*COF-Ru. a) Survey scan. b) N 1s. c) S 2p.

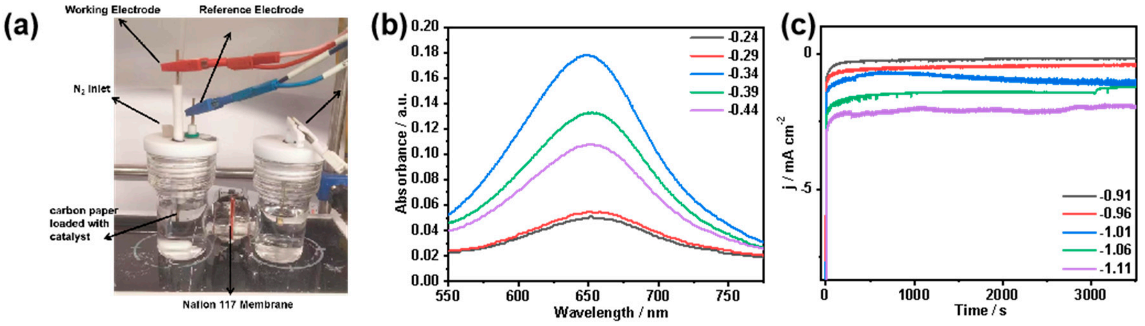

Figure S19. a) H-type cell setup for eNRR. b) UV–vis absorption spectra of working electrolyte after every one hour at 5 different voltages. c) Chronoamperometric curves of NSPC-Ru after every one hour at 5 different voltages.

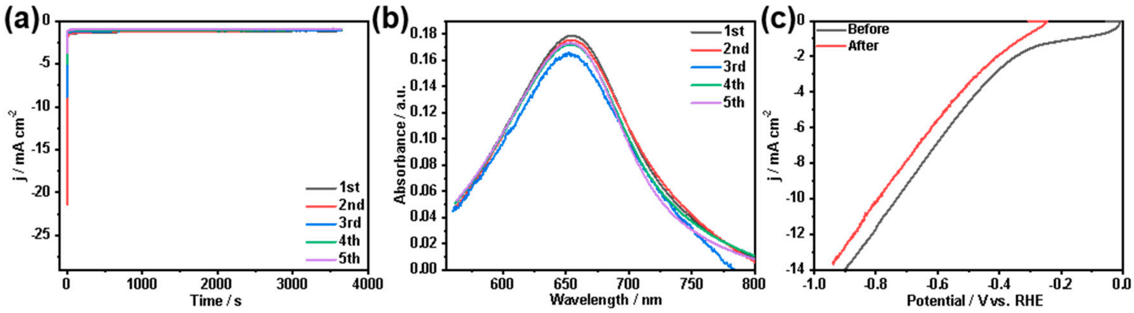

Figure S20. a) LSV curve before and after the test. b) UV–vis absorption spectra of working electrolyte after every one hour for 5 cycles. c) Chronoamperometric curves for NSPC-Ru after every one hour for 5 cycles at -0.34 V vs RHE.

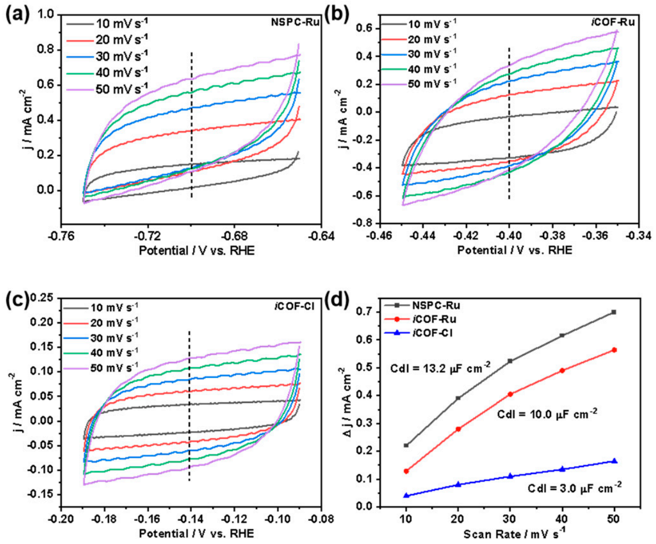

Figure S21. a, b, c) Double-layer capacitance obtained by CV for NSPC-Ru, *i*COF-Ru, and *i*COF-Cl. b) C_{dl} calculated by linear fitting.

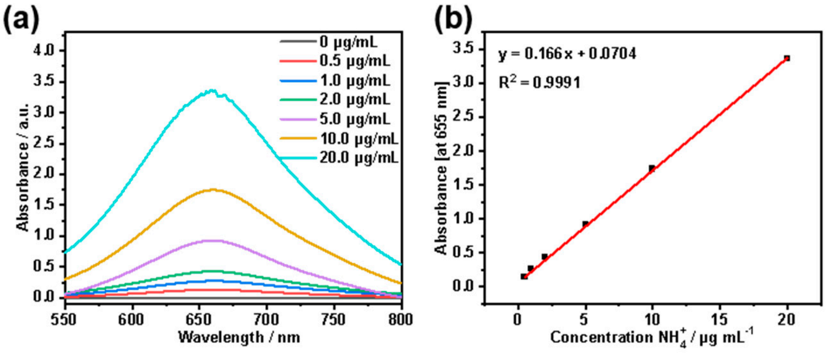

Figure S22. a) UV–vis absorption spectra. b) Corresponding calibration curve of colorimetric NH_4^+ method.

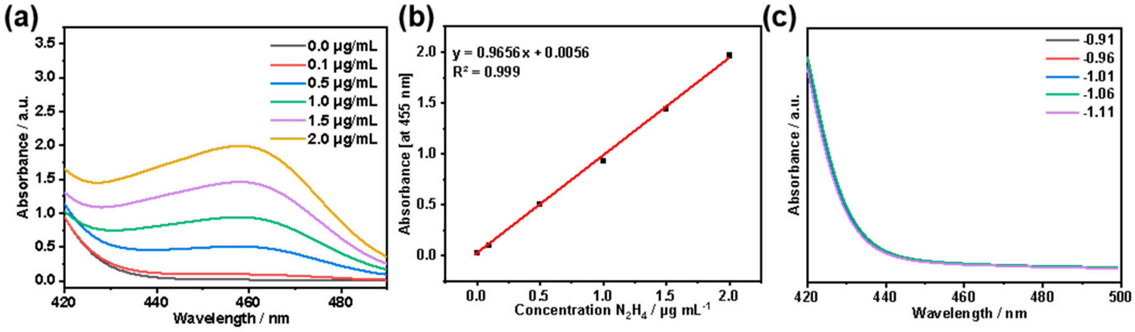

Figure S23. a) UV–vis absorption spectra. b) Corresponding calibration curve of colorimetric N_2H_4 method using Watt and Chrisp method in electrolytes. c) NSPC-Ru at different voltages via Watt and Chrisp method.

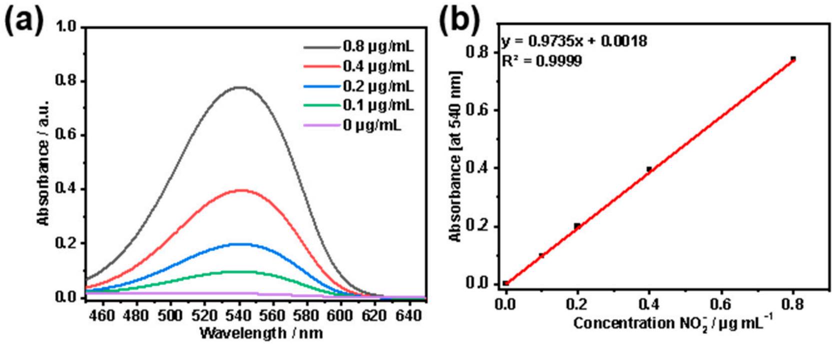

Figure S24. a) UV–vis absorption spectra. b) Corresponding calibration curve of colorimetric NO_2 method.

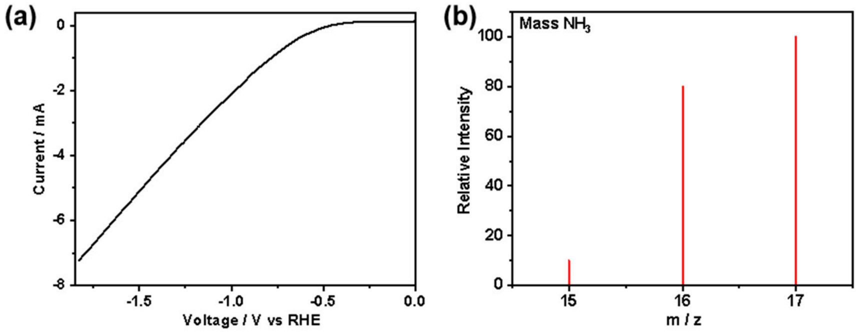

Figure S25. a) LSV calculated for online DEMS analysis. b) Standard mass spectra of NH_3 gas in NIST database.

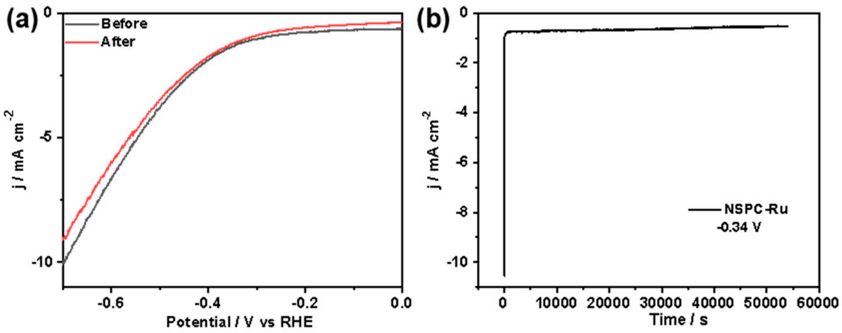

Figure S26. a) LSV curves of NSPC-Ru before and after long-term stability test. b) Long-term stability of NSPC-Ru.

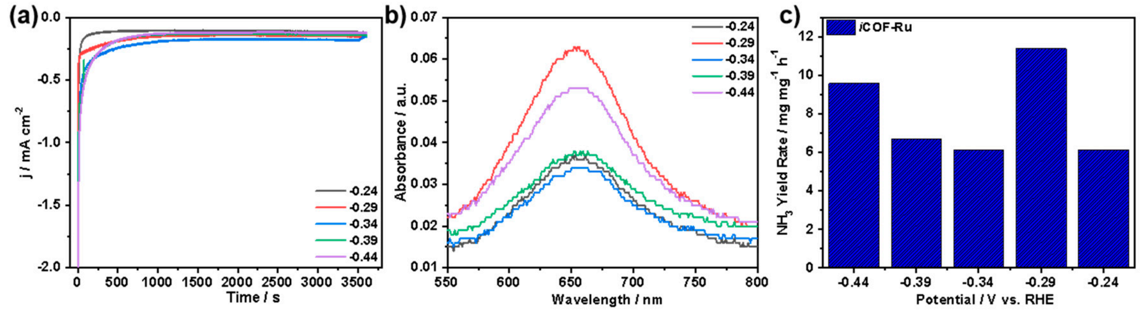

Figure S27. a) Chronoamperometric curves for NSPC-Ru after every one hour at 5 different voltages. b) UV–vis absorption spectra. c) NH3 yield rates for iCOF-Ru for eNRR.

Table S1. XPS analysis for *i*COF-Cl, *i*COF-Ru, and NSPC-Ru.

Name	C 1s Atomic (%)	N 1s Atomic (%)	S 2p Atomic (%)	Ru 3d Atomic (%)	O 1s Atomic (%)	Cl 2p Atomic (%)
*i*COF-Cl	89.31	6.88	3.1	-	-	0.7
*i*COF-Ru	78.97	4.08	0.93	6.32	9.70	
NSPC-Ru	62.79	1.15	0.42	5.03	30.61	

Table S2. Conductivity measurements at different temperature and relative humidity for *i*COF-Cl.

Temperature (^oC)	1000/T (k^{-1})	Log ($S\ cm^{-1}$)	Relative Humidity (%)	Conductivity ($S\ cm^{-1}$)
25.2	3.323	-3.276	57	$5.02*10^{-3}$
30.2	3.297	-3.172	68.9	$6.03*10^{-3}$
35	3.245	-3.046	75.3	$6.6*10^{-3}$
40	3.193	-2.967	84.3	$9.32*10^{-3}$
50	3.094	-2.844	98	$1.219*10^{-2}$
60	3.001	-2.672		

Table S3. Comparison table for conductivity with different materials.

Materials	Ionic condivity (S cm^{-1})	Test conditions	Reference
BUT-8(Cr)A	1.27×10^{-1}	80 C 100 % RH	*Nat Energy*, **2017**, *2*, 877-883
UiO-66(SO_3H)$_2$	8.4×10^{-2}	80 C 90 % RH	*Angew. Chem. Int. Ed.*, **2015**, *54*, 5142-5146
PCMOF-10	3.55×10^{-2}	60 C 95 % RH	*J. Am. Chem. Soc.*, **2015**, *137*, 913–918
PCMOF21 /2	2.1×10^{-2}	85 C 90 % RH	*J. Am. Chem. Soc.*, **2018**, *140*, 1077–1082
*i*COF-Cl	1.219×10^{-2}	60 C 98 % RH	This work
Im–Fe–MOF	1.21×10^{-2}	60 C 98 % RH	*J. Am. Chem. Soc.*, **2017**, *139*, 6183–6189
PCMOF-5	4×10^{-3}	62 C 98 % RH	*J. Am. Chem. Soc.*, **2013**, *135*, 1193–1196
CB[6]·1.2H_2SO_4·6.4H_2O	1.3×10^{-3}	25 C 98 % RH	*Angew. Chem. Int. Ed.*, **2011**, *50*, 7870-7873
MFM-500(Ni)	4.5×10^{-4}	25 C 98 % RH	*J. Am. Chem. Soc.*, **2016**, *138*, 6352–6355
$(Me_2NH_2)2[Li_2Zr(C_2O_4)_4]$	3.9×10^{-5}	17 C 67 % RH	*J. Am. Chem. Soc.*, **2015**, *137*, 6428–6431
H2TDPP·(DMF)6·(THF)5	3.4×10^{-6}	27 C 97 % RH	*Cryst. Growth Des.*, **2016**, *16*, 5831–5835

Table S4. Comparison of different catalysts in electrochemical nitrogen reduction reaction.

Catalyst	Electrolyte	Potential	FE (%)	NH_3 yield rate	Reference
Ru_2P-rGO	0.1 M HCl	-0.05 V vs. RHE	13.04	32.8 μg h^{-1} mg_{cat}^{-1}	*J. Mater. Chem. A*, **2020**, *8*, 77-81
Rh_2Sb RNRs	0.5 M Na_2SO_4	-0.45 V vs. RHE	1.5	228.85 μg h^{-1} mg_{Rh}^{-1}	*Angew. Chem. Int. Ed.*, **2020**, *59*, 8066-8071
Fe-ReS_2@N-CNF	0.1M Na_2SO_4	-0.2V vs. RHE	12.3	80.4 μg h^{-1} mg_{cat}^{-1}	*Adv. Funct. Mater.*, **2020**, *30*, 1907376
Rh-Se NCs	0.1 M HCl	-0.1V vs. RHE	13.3	175.6 μg h^{-1} mg_{cat}^{-1}	*Adv. Mater.*, **2020**, *32*, 2001267
a-Au/CeO_x–RGO	0.1 M HCl	−0.2 V vs. RHE	10.1	8.3 μg h^{-1} mg_{cat}^{-1}	*Adv. Mater.*, **2017**, *29*, 1700001
RuSAs/N-C	0.05 M H_2SO_4	-0.2 V vs. RHE	29.6	120.9 μg h^{-1} mg_{cat}^{-1}	*Adv. Mater.*, **2018**, *30*, 1803498
Ru@ZrO_2/NC	0.05 M HCl	−0.21 V vs. RHE	15	3.665 μg h^{-1} mg_{cat}^{-1}	*Chem*, **2019**, *5*, 204-214
B-Doped graphene	0.05 M H_2SO_4	−0.5V vs. RHE	10.8	54.88 μg h^{-1} mg_{cat}^{-1}	*Joule*, **2018**, *2*, 1610-1622
Rh nanosheet	0.1 M KOH	-0.2 vs. RHE	0.217	23.88 μg h^{-1} mg_{cat}^{-1}	*J. Mater. Chem. A*, **2018**, *6*, 3211-3217
Ru SAs/N-C	0.05 M H_2SO_4	-0.2 vs. RHE	29.6	120.9 μg h^{-1} mg_{cat}^{-1}	*Adv. Mater.*, **2018**, *30*, 1803498
Boron rich amorphous COF/NC	0.1 M KOH	-0.2 vs. RHE	45.3	12.53 μg h^{-1} mg_{cat}^{-1}	*Nat. Commun.*, **2019**, *10*, 3898
CuII–MOF on carbon cloth	1 M Na_2SO_4	0.4 vs. RHE	11.9	24.7 μg h^{-1} mg_{cat}^{-1}	*Chem. Commun.*, **2019**, *55*, 10170-10173
LaFeO-Ru	0.1 M K_2SO_4	-0.7 vs. RHE	56.9	137.5 μg h^{-1} mg_{cat}^{-1}	*Small*, **2023**, *19*, 2208102
NSPC-Ru	0.1 M K_2SO_4	-0.34 vs. RHE	13.2	32.0 μg h^{-1} mg_{cat}^{-1}	This Work
